# Supplementary material for: Water induced ultrathin Mo2C nanosheets with high-density grain boundaries for enhanced hydrogen evolution
Source: Nat Commun. 2022 Nov 24;13:7225. doi: 10.1038/s41467-022-34976-1 (PMC9700844; doi:10.1038/s41467-022-34976-1)
Supplement: Supplementary file 1 — Supplementary Information [file 41467_2022_34976_MOESM1_ESM.pdf]

Supplementary Information

Water Induced Ultrathin Mo_2C Nanosheets with High-Density Grain Boundaries for Enhanced Hydrogen Evolution

Yang *et al.*

Supplementary Figures

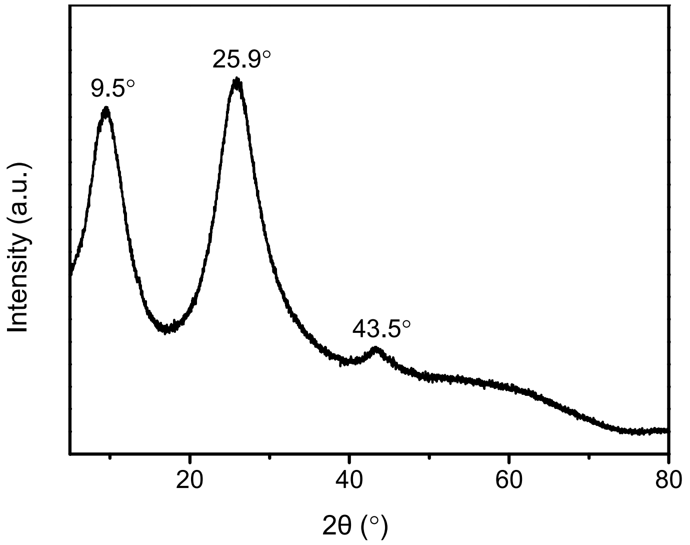

Supplementary Fig. 1. XRD pattern of MoO_3/RGO intermediate. The sharp peak around 9.5° is attributed to GO with an interlayer spacing of 0.93 nm, which is attributed to the introduction of oxygen-containing groups into the graphite lattice. The peaks at 25.9° and 43.5° are assigned to the (002) and (100) planes of 2H phase of graphite with *d*-spacing of 0.34 nm. No peaks derived from MoO_3 are detected[1], clearly showing the amorphous nature of MoO_3.

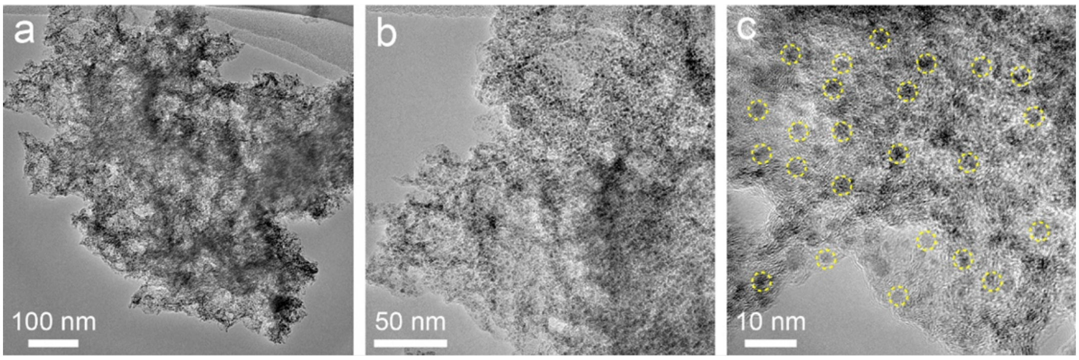

Supplementary Fig. 2. TEM images of Mo_2C/NG. The Mo_2C NPs are highlighted by yellow dotted circles.

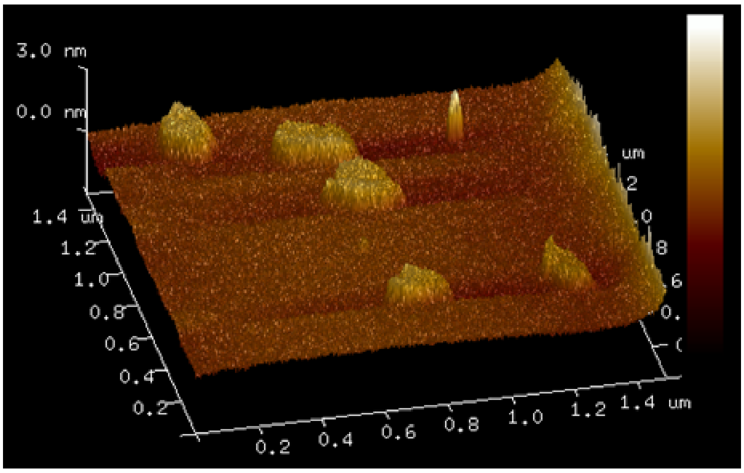

Supplementary Fig. 3. The corresponding 3D topography of Fig. 2c in the main text.

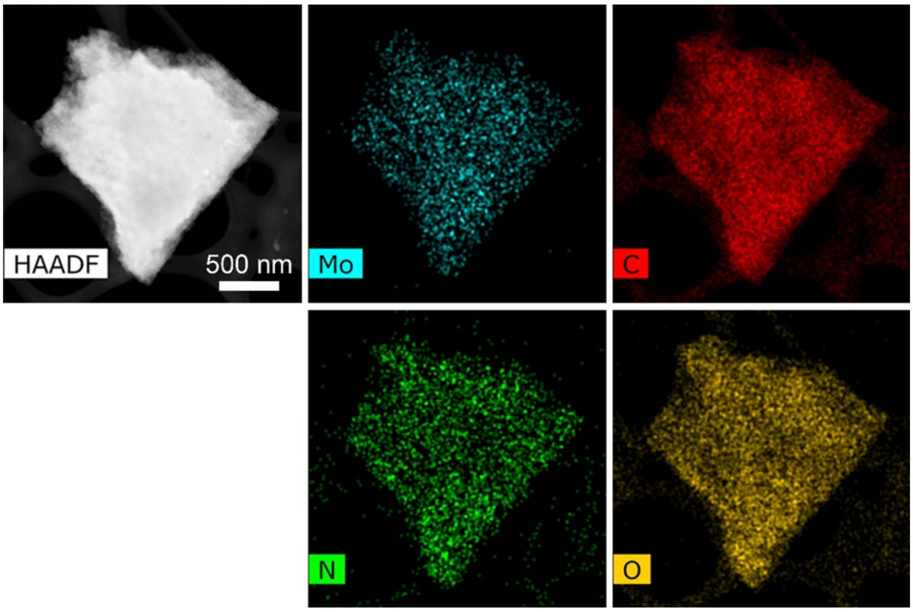

Supplementary Fig. 4. HAADF-STEM image of H-Mo_2C/NG and the corresponding EDS mapping images of Mo, C, N, and O elements. The spatial distribution of Mo, C, and N elements reveals the uniform growth of Mo_2C NSs over NG matrix. Oxygen element can also be observed due to the surface oxidation of Mo_2C nanocrystals.

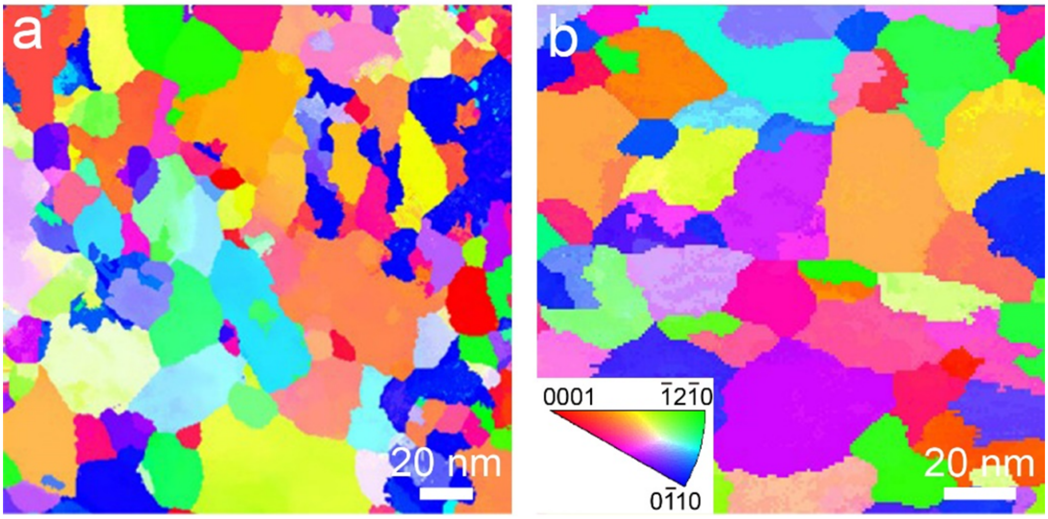

Supplementary Fig. 5. Inverse pole figure (IPF) images from PED analysis. **a** H-Mo_2C/NG-13.52 and **b** H-Mo_2C/NG-30.98.

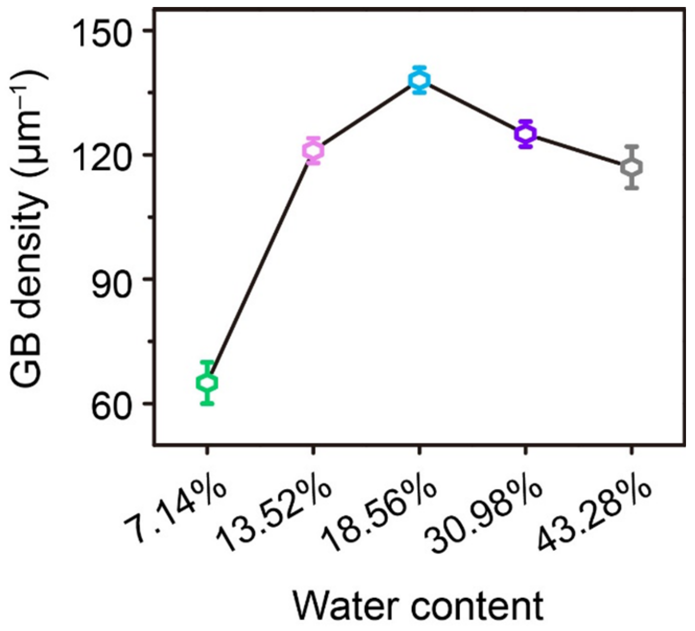

Supplementary Fig. 6. Statistical GB density of Mo_2C NSs in Mo_2C hybrids. The GB density was calculated by dividing the total GB length with the total sample area.

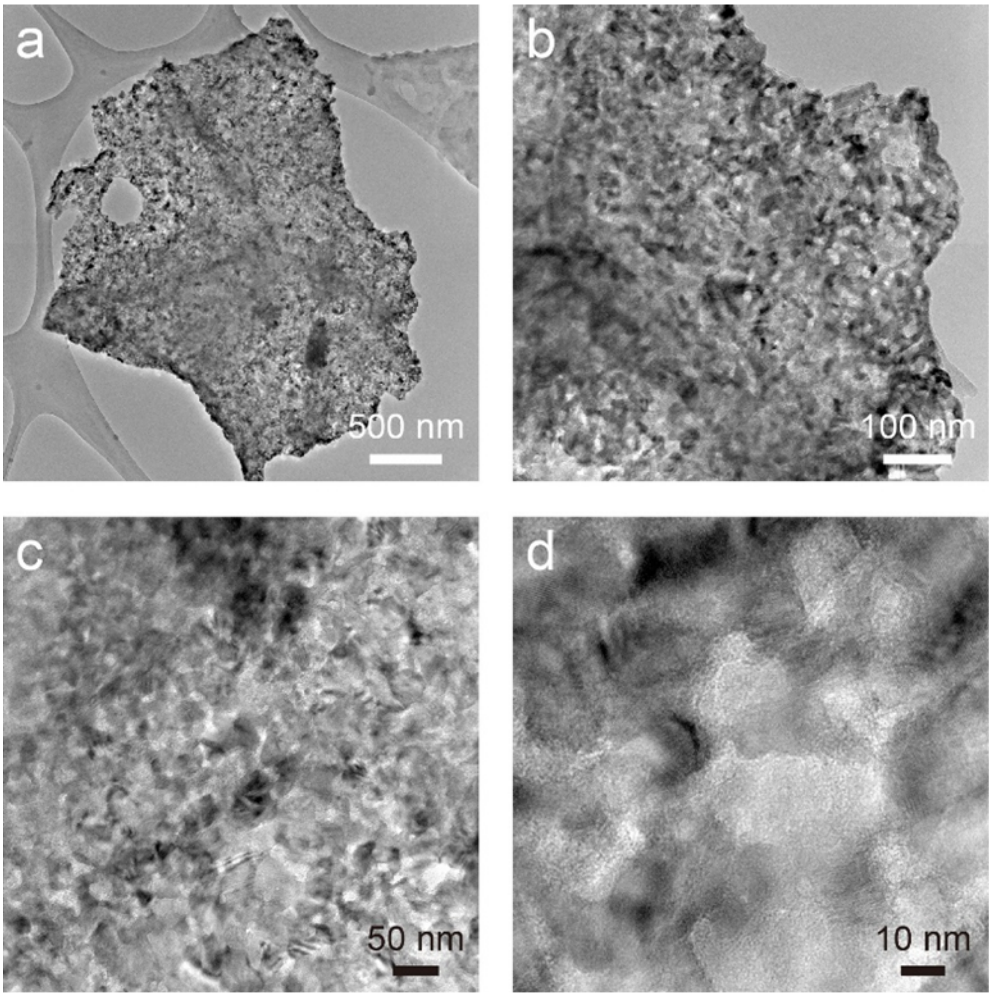

Supplementary Fig. 7. TEM images of H-Mo_2C/NG-7.14.

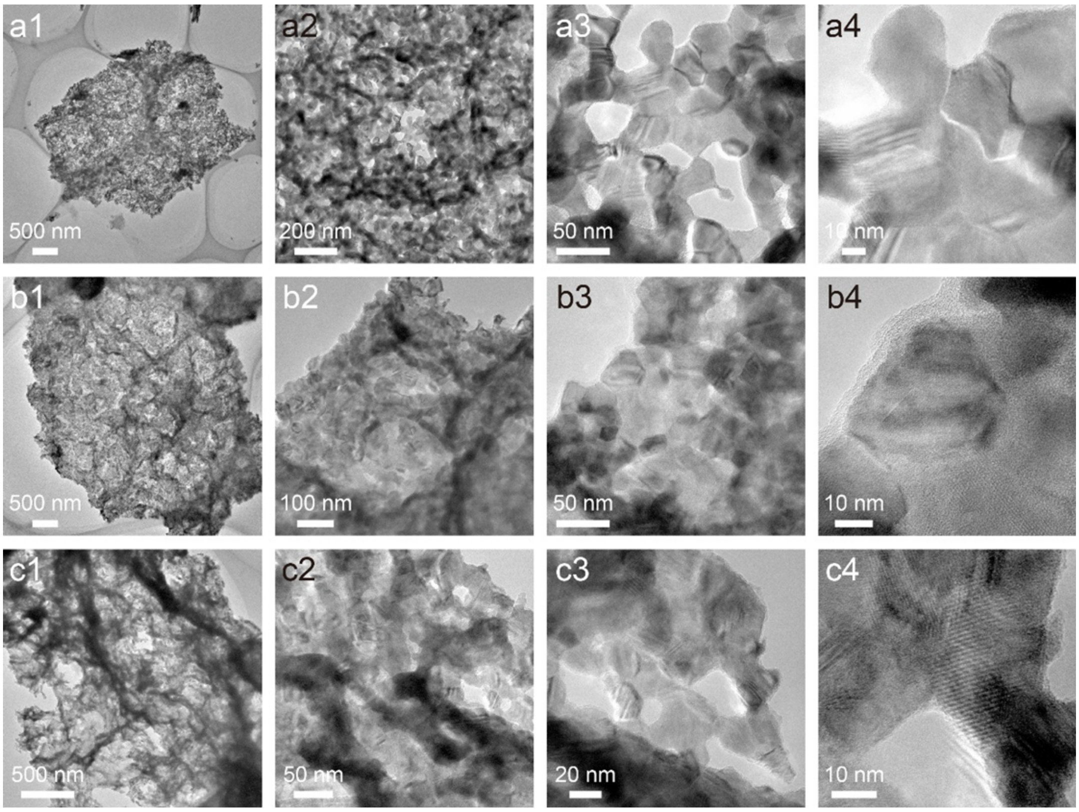

Supplementary Fig. 8. TEM images of Mo_2C hybrids. a1-a4 H-Mo_2C/NG-13.52, **b1-b4** H-Mo_2C/NG, and **c1-c4** H-Mo_2C/NG-30.98.

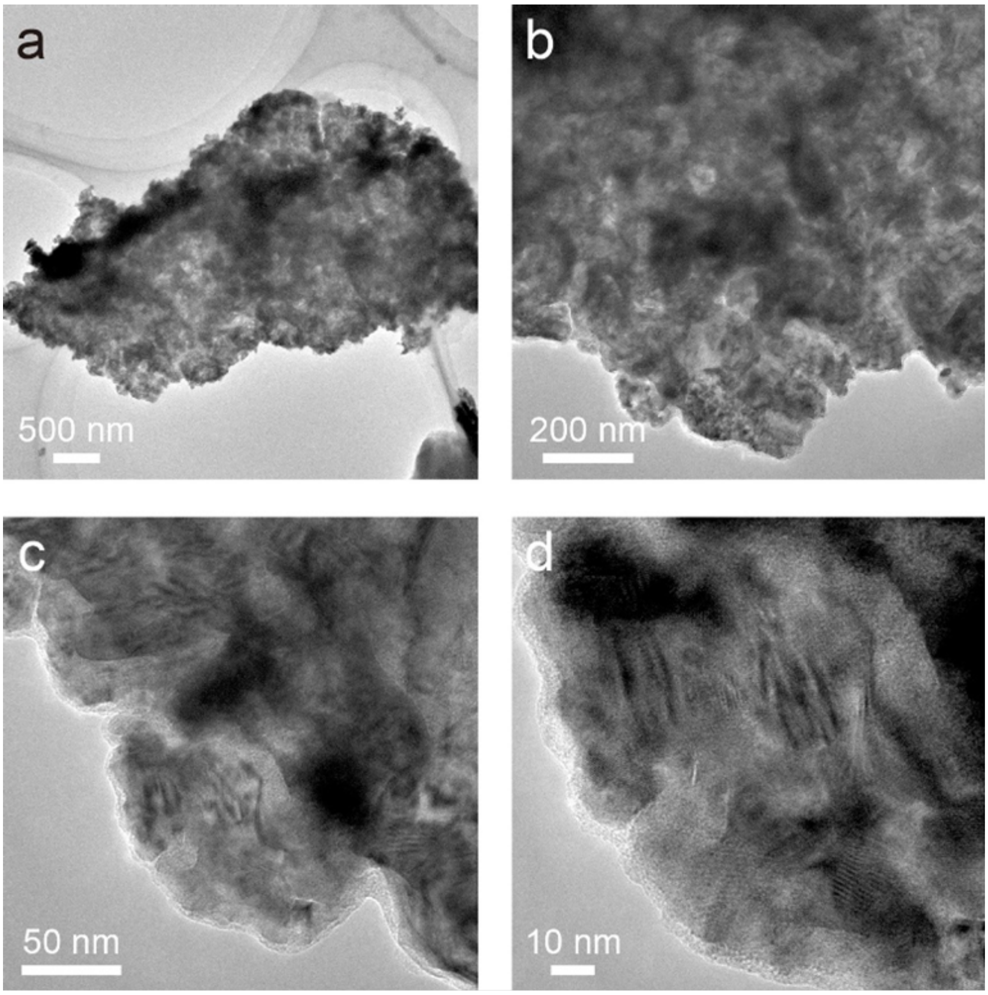

Supplementary Fig. 9. TEM images of H-Mo_2C/NG-43.28.

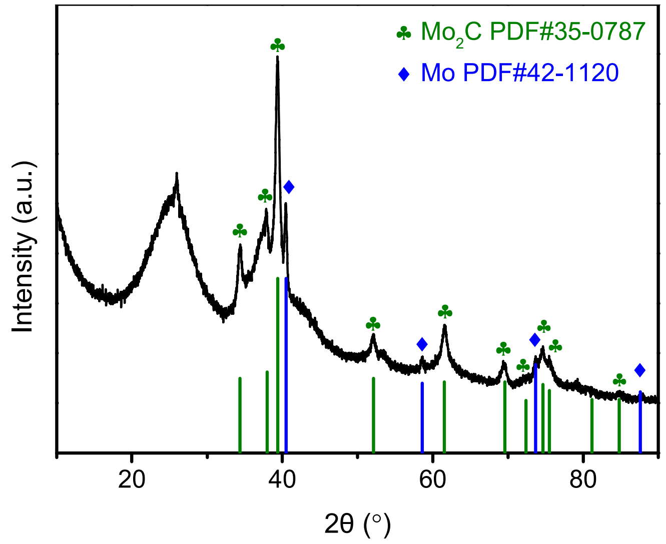

Supplementary Fig. 10. XRD pattern of Mo-Mo_2C heterostructures on RGO. The XRD pattern reveal the formation of compositions of Mo_2C nanocrystals and metallic Mo with the same procedure as H-Mo_2C/NG without NH_3.

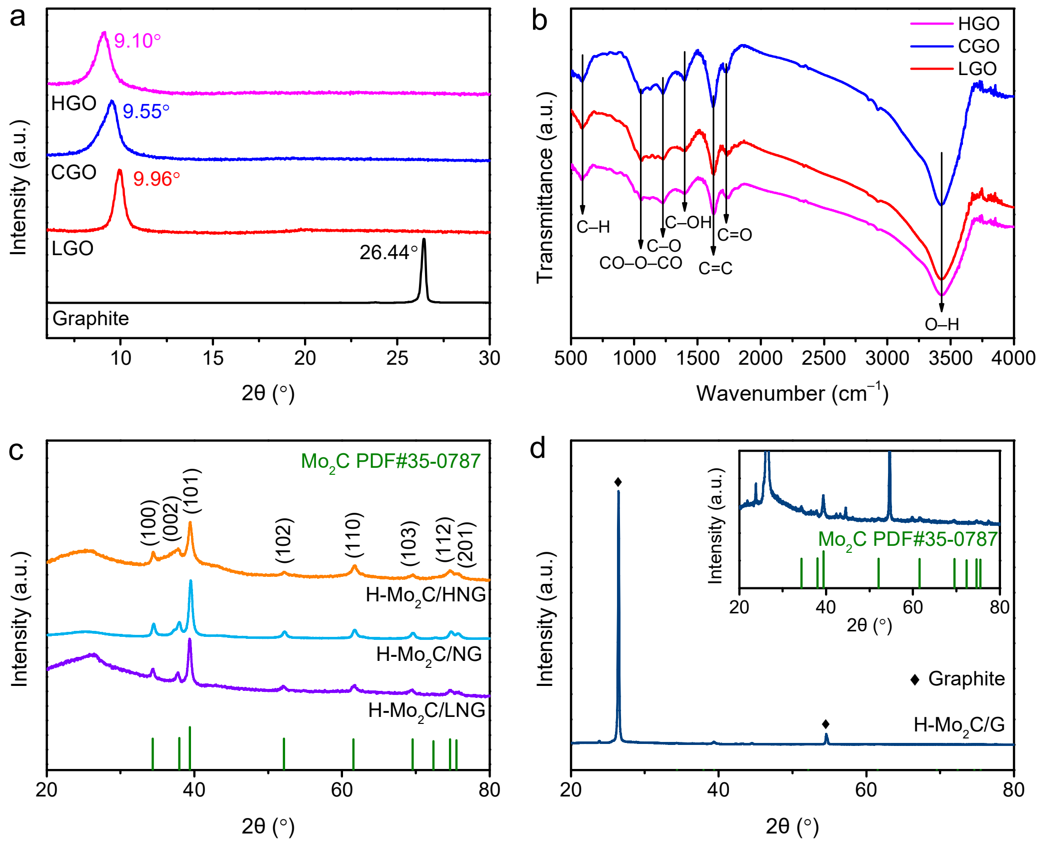

Supplementary Fig. 11. Characterization results. a XRD patterns of high oxidation degree of GO (HGO)[2], common GO with medium oxidation degree (CGO) used to construct the H-Mo_2C/NG, low oxidation degree of GO (LGO)[3], and graphite. The graphite has a sharp diffraction (002) peak at $2\theta = 26.44°$ with an interlayer spacing of 3.37 Å calculated form Bragg equation. After oxidation and exfoliation of graphite, HGO, CGO, and LGO exhibit the characteristic (001) peaks at $2\theta = 9.10°$, 9.55°, and 9.95° with the corresponding interlayer spacing of 9.710, 9.254, and 8.883 Å , respectively, indicating that the order of oxidation levels is HGO>CGO>LGO. **b** Fourier transform infrared (FTIR) spectra. **c** XRD patterns of H-Mo_2C/HNG, H-Mo_2C/NG, and H-Mo_2C/LNG. **d** XRD pattern of H-Mo_2C/G. H-Mo_2C/HNG, H-Mo_2C/LNG, and H-Mo_2C/G were synthesized under identical synthesis conditions with H-Mo_2C/NG, in which CGO was replaced by HGO, LGO, and graphite respectively.

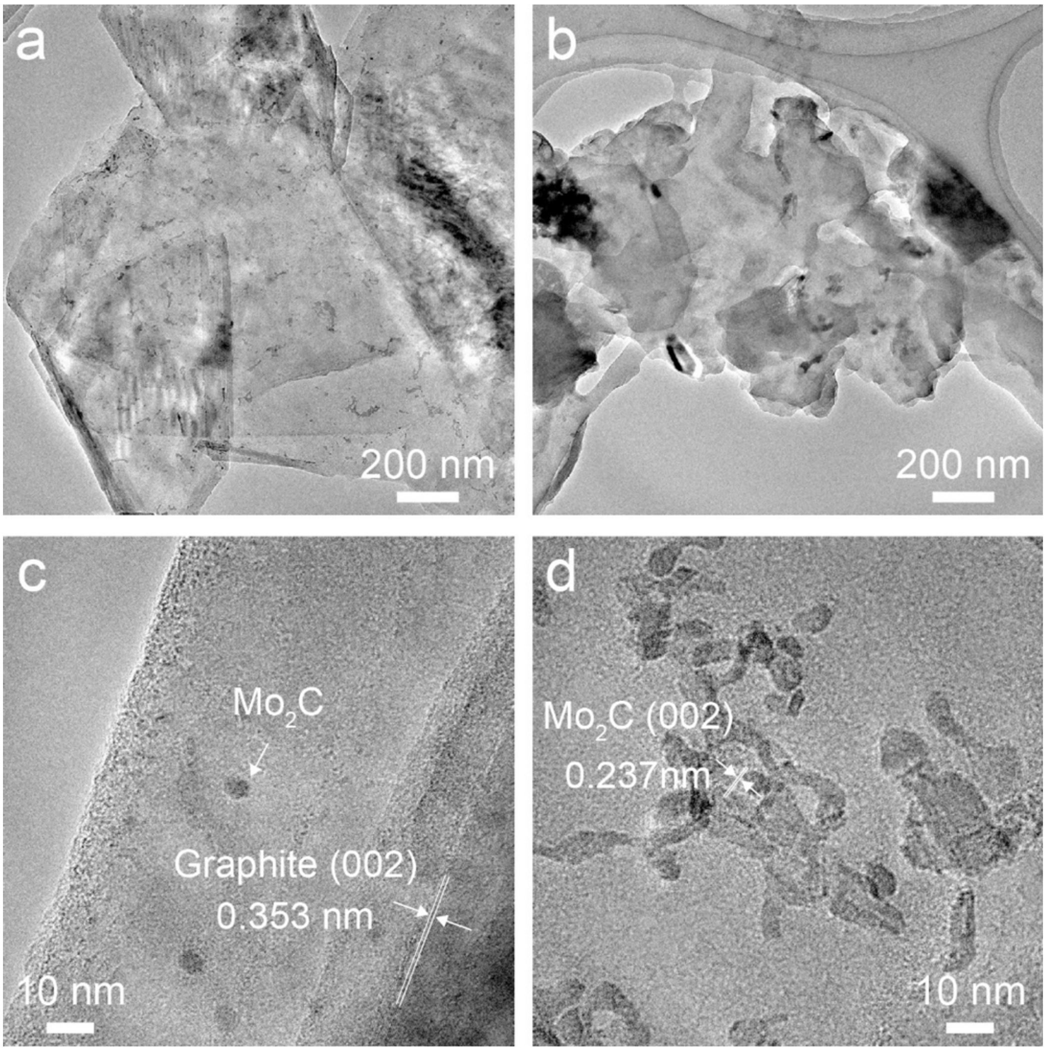

Supplementary Fig. 12. TEM characterization of H-Mo_2C/G.

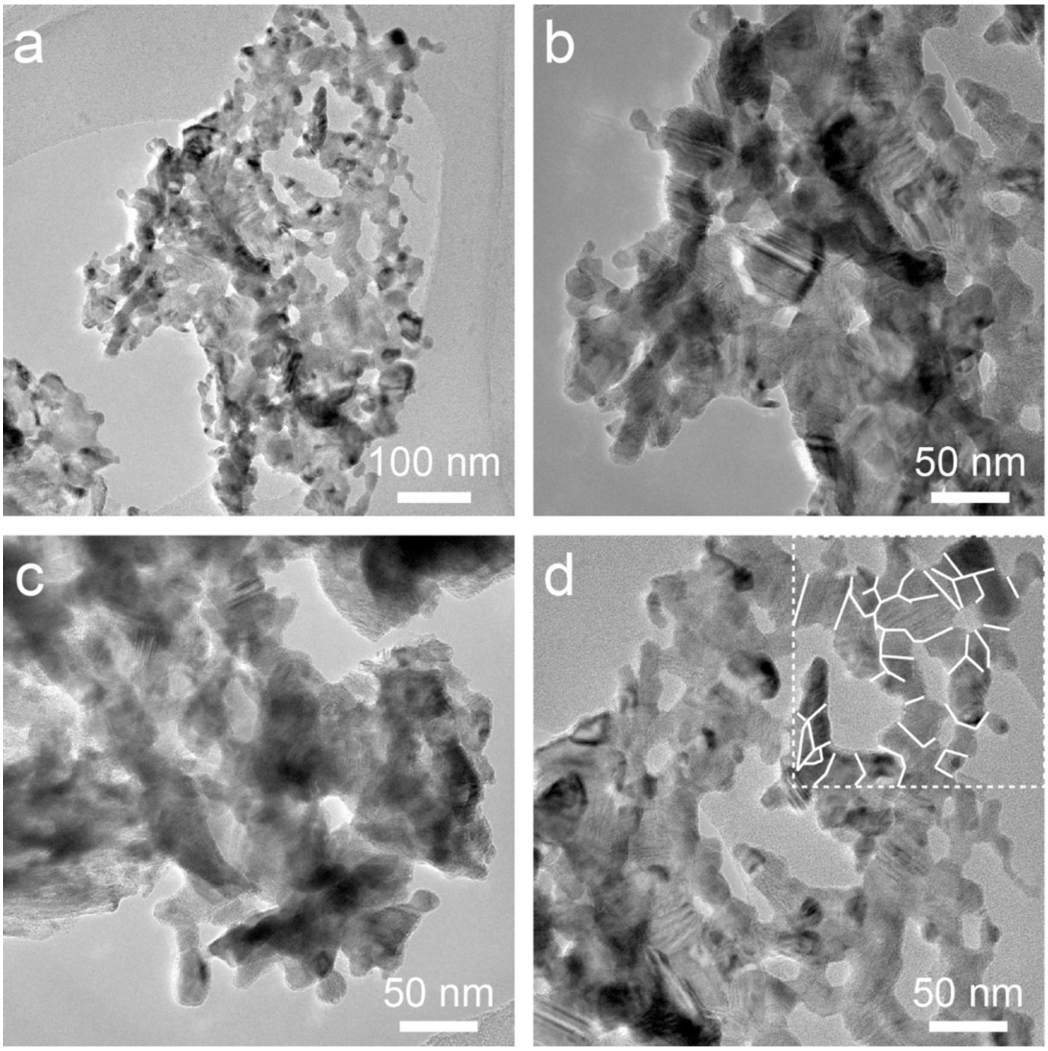

Supplementary Fig. 13. TEM characterization of H-Mo_2C/LNG. The solid lines in boxed region in **d** point out the irregular GBs.

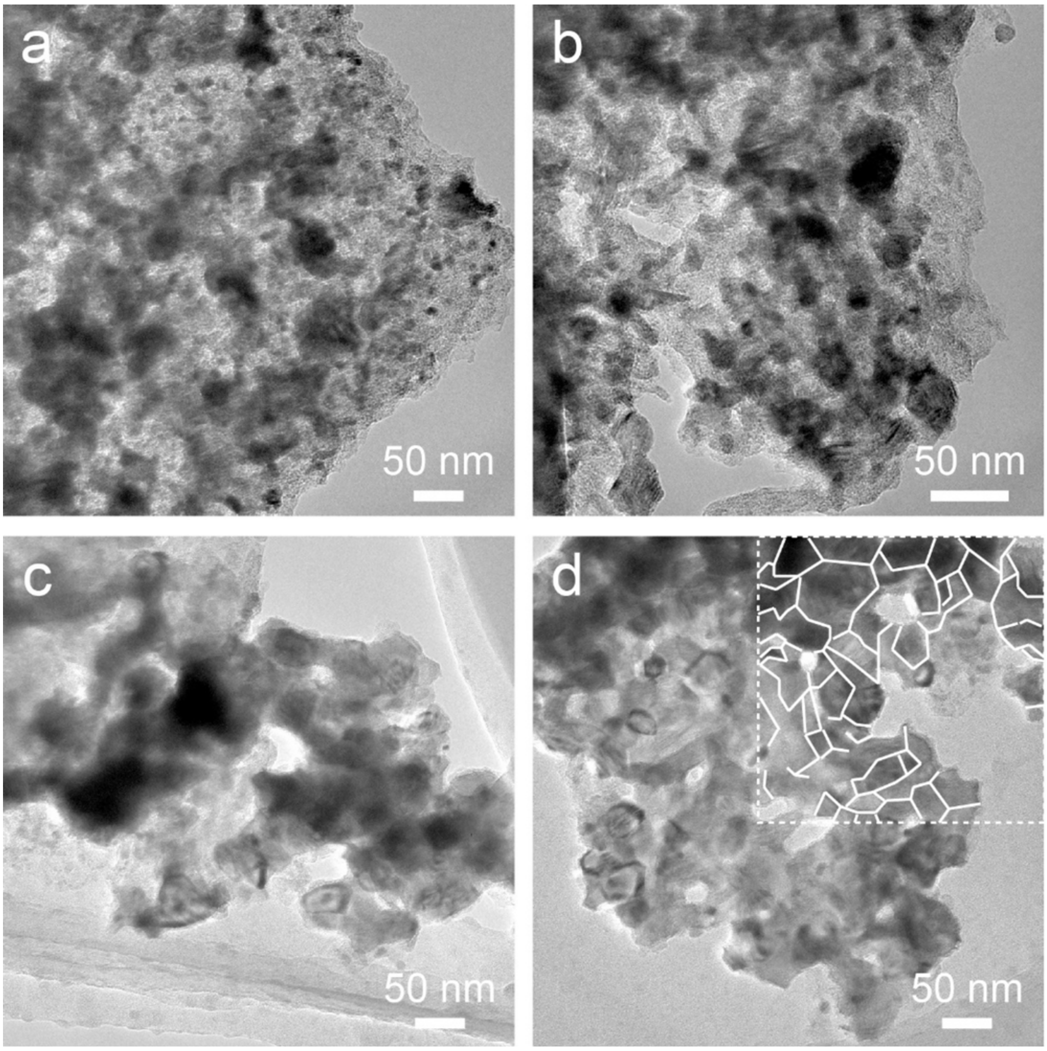

Supplementary Fig. 14. TEM characterization of H-Mo_2C/HNG. The solid lines in boxed region in **d** point out the irregular GBs.

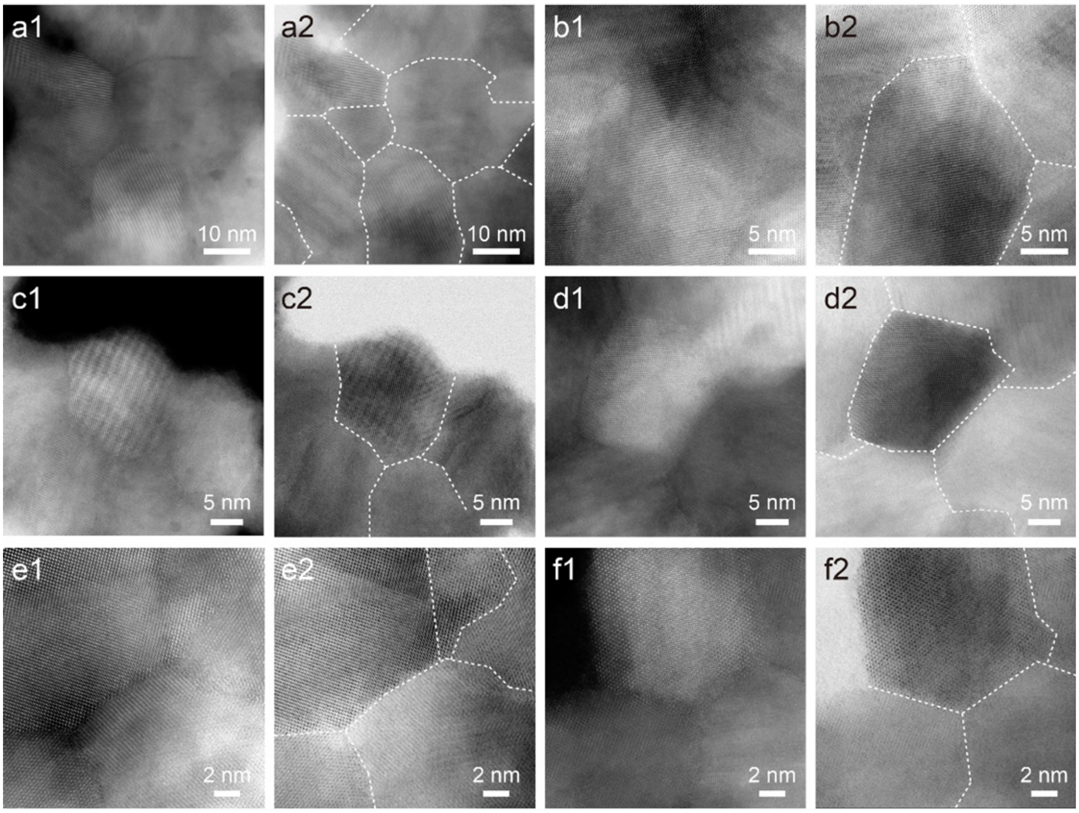

Supplementary Fig. 15. Atomic-resolution STEM images of Mo_2C NSs in H-Mo_2C/NG. a1-f1 HAADF-STEM and **a2-f2** bright-field STEM (BF-STEM) images, the white dashed lines point out the irregular GBs.

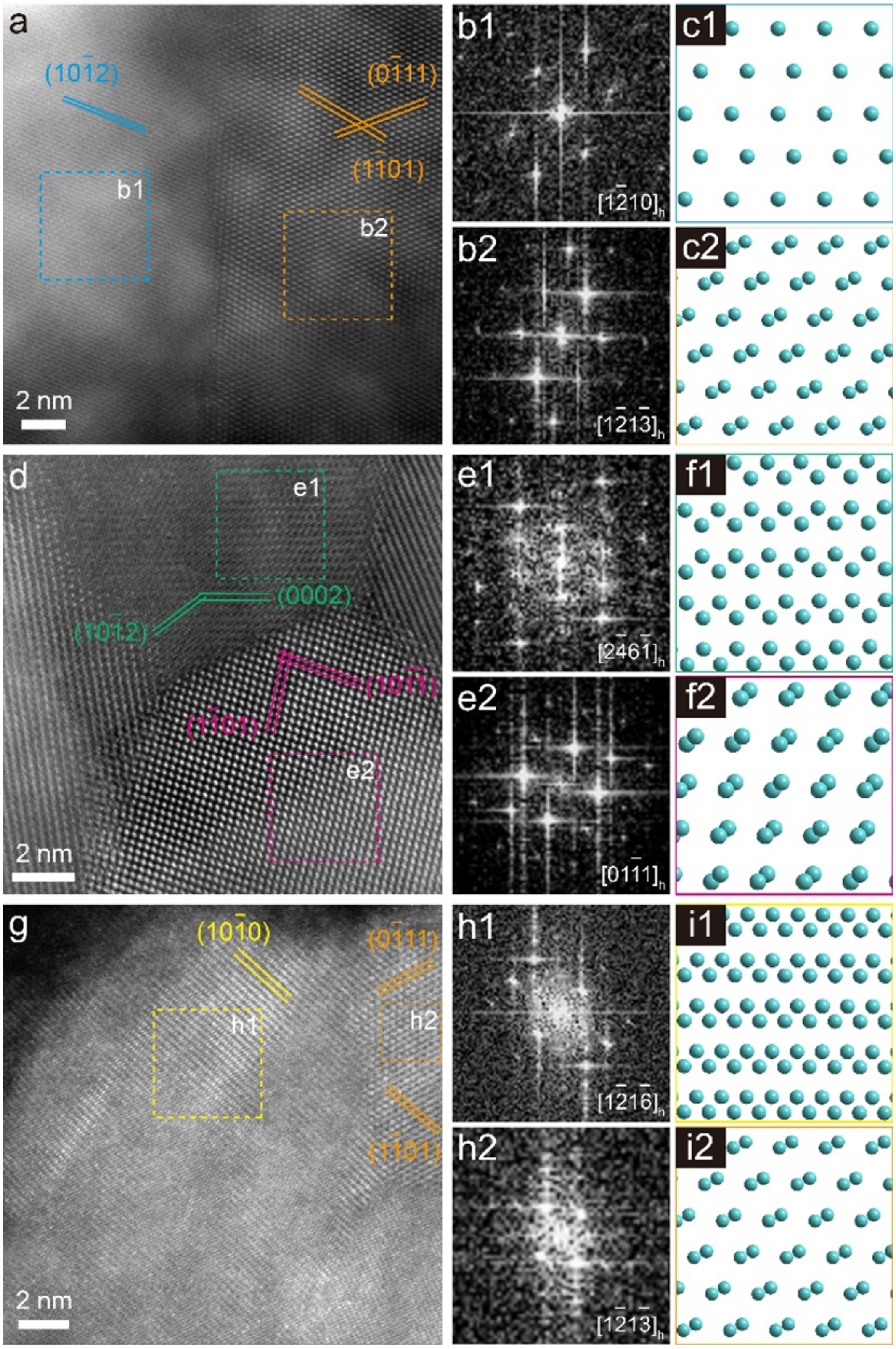

Supplementary Fig. 16. Local atomic structure of hcp/hcp GBs on Mo_2C NSs in H-Mo_2C/NG. a HAADF-STEM image taken along the [1$\bar{2}$10]$_h$/[1$\bar{2}$1$\bar{3}$]$_h$ direction. **b1**, **b2** FFT patterns taken from the corresponding royal blue and orange dotted squares in

a, and **c1**, **c2** the corresponding projected supercell models with hcp Mo_2C structures. **d** HAADF-STEM image taken along the $[2\bar{4}6\bar{1}]_h/[01\bar{1}1]_h$ direction. **e1**, **e2** FFT patterns taken from the corresponding dark green and dark pink dashed squares in **d**, and **f1**, **f2** the corresponding projected supercell models with hcp Mo_2C structures. **g** HAADF-STEM image taken along the $[1\bar{2}1\bar{6}]_h/[1\bar{2}1\bar{3}]_h$ direction. **h1**, **h2** FFT patterns taken from the corresponding yellow and orange dashed squares in **g**, and **i1**, **i2** the corresponding projected supercell models with hcp Mo_2C structures. The cyan balls represent Mo atoms.

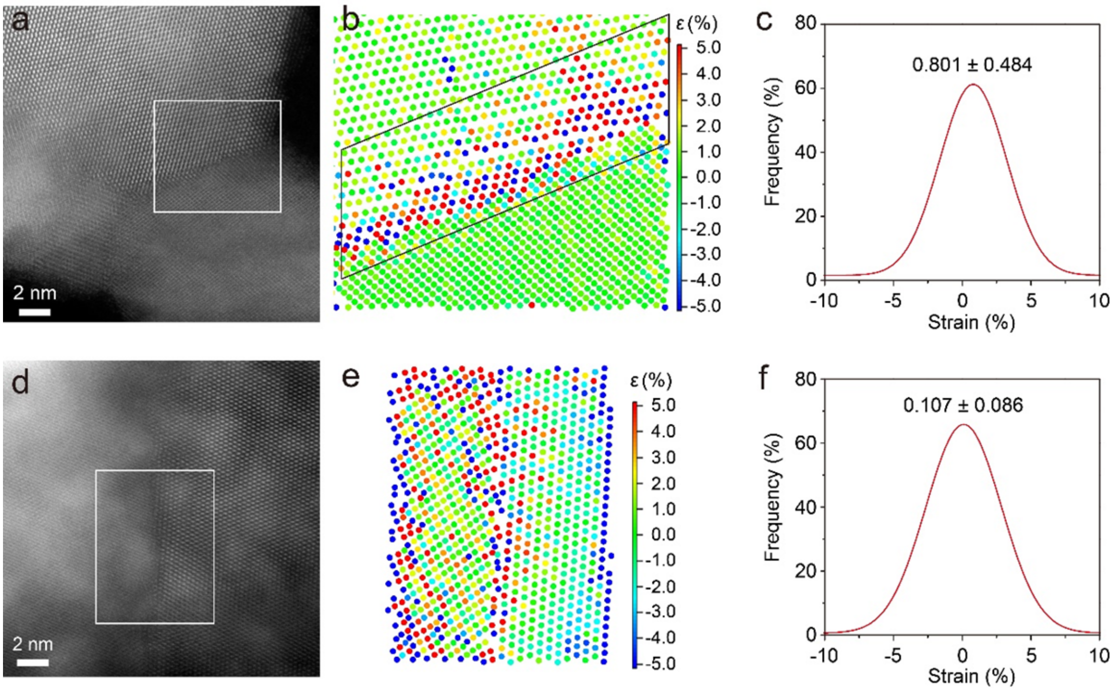

Supplementary Fig. 17. HADDF-STEM images and strain behaviours of Mo_2C NSs in H-Mo_2C/NG. **a** HADDF-STEM image. **b** Lattice distortion determined from the white-boxed region in **a**. **c** Quantitative distribution of shear strain for atoms in the black-boxed region in **b**. **d** HADDF-STEM image. **e** Lattice distortion determined from the white-boxed region in **d**. **f** Quantitative distribution of shear strain for atoms in **e**. The atoms are coloured according to strain scale, where colours with positive values represent tensile strain and with negative values represent compressive strain.

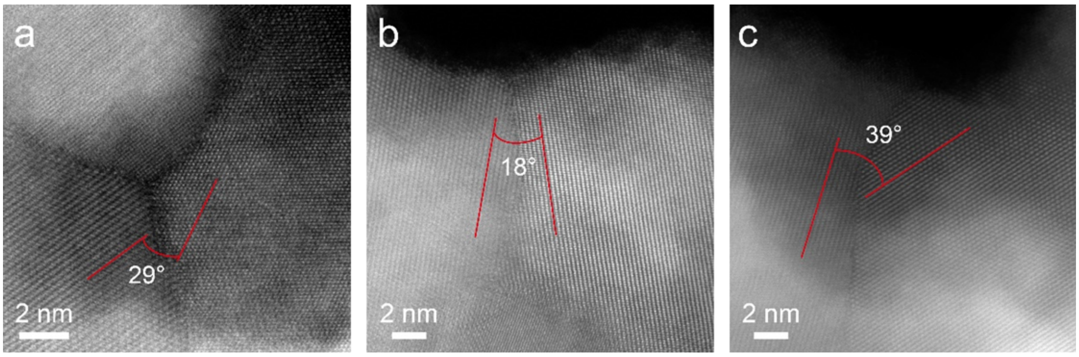

Supplementary Fig. 18. Atomic-resolution HAADF-STEM images of GBs on Mo_2C NSs in H-Mo_2C/NG.

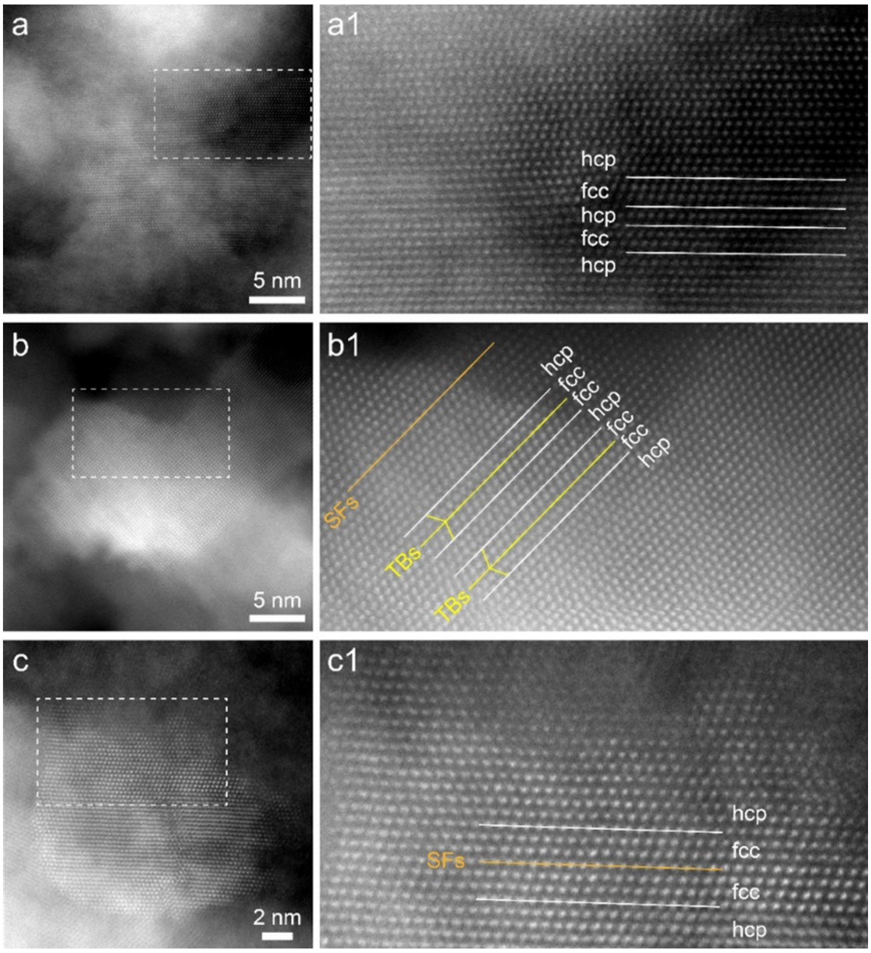

Supplementary Fig. 19. Local atomic structure of fcc/hcp GBs on Mo_2C NSs in H-Mo_2C/NG. a-c HAADF-STEM images taken along the $[110]_f/[11\bar{2}0]_h$ direction. **a1-c1** Corresponding enlarged HAADF-STEM images of the rectangular regions in **a-c**. The fcc/hcp GBs are marked with white solid lines. Typical defects including SFs and TBs are marked with orange and yellow lines, respectively.

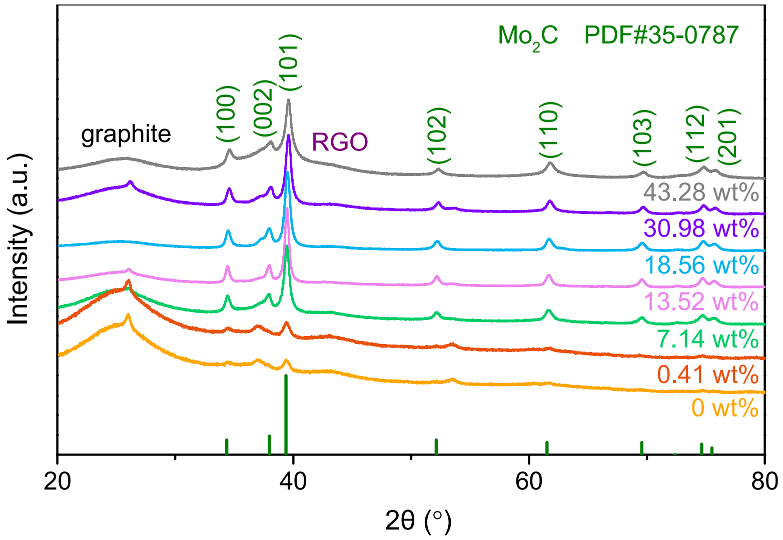

Supplementary Fig. 20. XRD patterns of Mo_2C hybrids. The peaks near 2θ = 26° and 43° are respectively assigned to the (002) and (100) reflections of graphene. And the weak peaks at 37° and 53° are considered to be the ($\bar{2}$11) and ($\bar{2}$22) planes of MoO_2 (PDF#32-0671), respectively. With the increase of water content, Mo_2C hybrids exhibit gradually enhanced diffraction peaks, which demonstrates that water promotes the morphological evolution of Mo_2C during carbonization process. Furthermore, the shift of peak positions for H-Mo_2C/NG and Mo_2C/NG is negligible, suggesting that the mean lattice distortion of the chemically connected Mo_2C NSs is almost neutralized. This result could be attributed to the anisotropic connection of each grain, which results in the mutual cancellation of stretched and suppressed defects, consistent with HADDF-STEM strain analyses. Mo_2C nanocomposites obtained by the anhydrous carburization process contain a small amount of MoO_2. However, as water content retained in MoO_3/RGO intermediate above 7.14 wt%, the molybdenum oxide peaks in Mo_2C hybrids prepared by the water-assisted carbothermal reaction are ignorable, indicative of a successful chemical conversion of amorphous MoO_3 into crystalline Mo_2C. These results also demonstrate that water promotes the formation of Mo_2C to a certain extent.

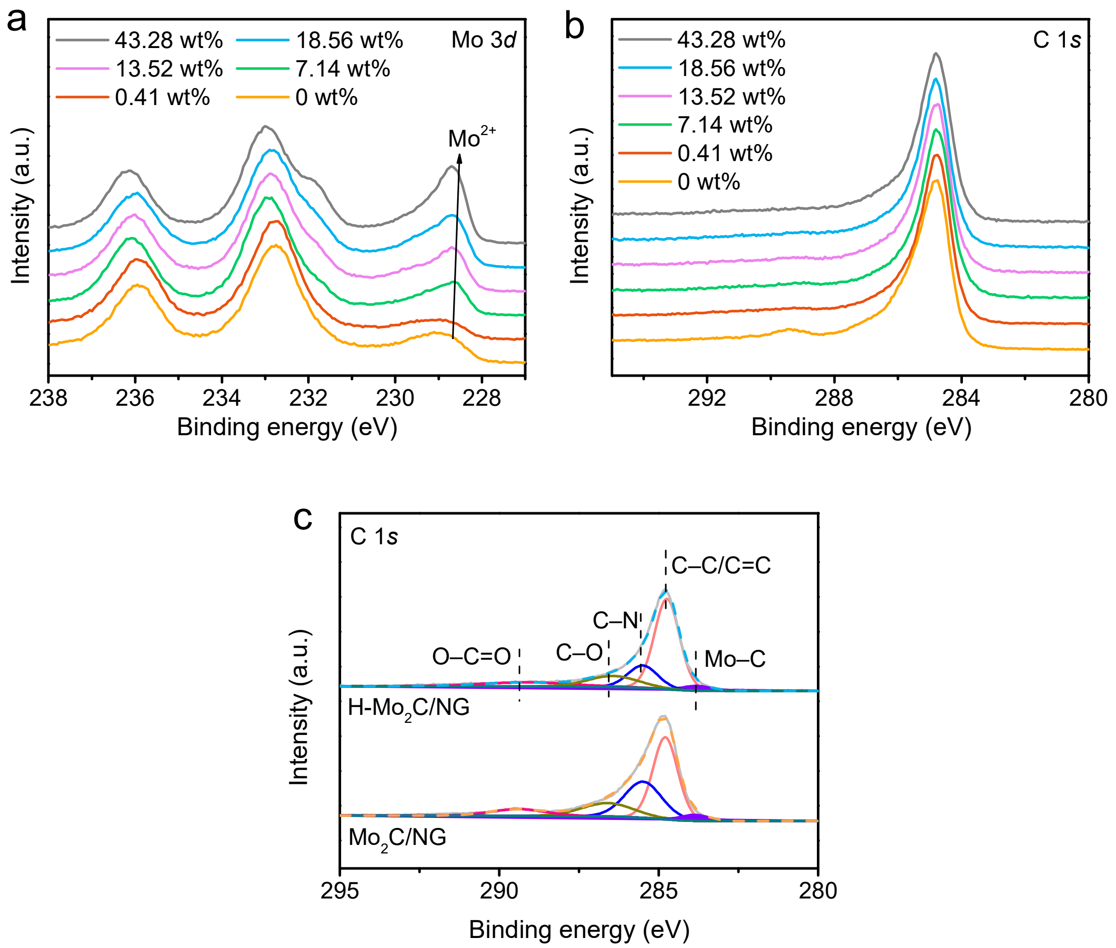

Supplementary Fig. 21. High resolution XPS spectra. a Mo 3*d* and **b** C 1*s* XPS spectra of Mo_2C hybrids. **c** High resolution C 1*s* XPS spectra of H-Mo_2C/NG and Mo_2C/NG. The atomic percentages of Mo^{3+} in H-Mo_2C/NG and Mo_2C/NG are 10.01 at% and 8.83 at% (Fig. 4b), respectively. Although the introduction of nitrogen atoms into Mo_2C is beneficial for electrocatalytic HER, the nitrogen species in Mo_2C have very little effect on HER activity[4-6]. The peaks of Mo^{2+} species shift to lower binding energy, accompanied by a significant increase of peak intensity, indicating that water promotes the formation of Mo_2C and simultaneously regulates the electronic structure. The C 1*s* spectra of H-Mo_2C/NG and Mo_2C/NG are similar and can be deconvoluted into five individual peaks of Mo–C (284.1 eV), C–C/C=C (284.8 eV), C–N (285.6 eV), C–O (286.5 eV), and O=C–O (289.6 eV) bonds[7].

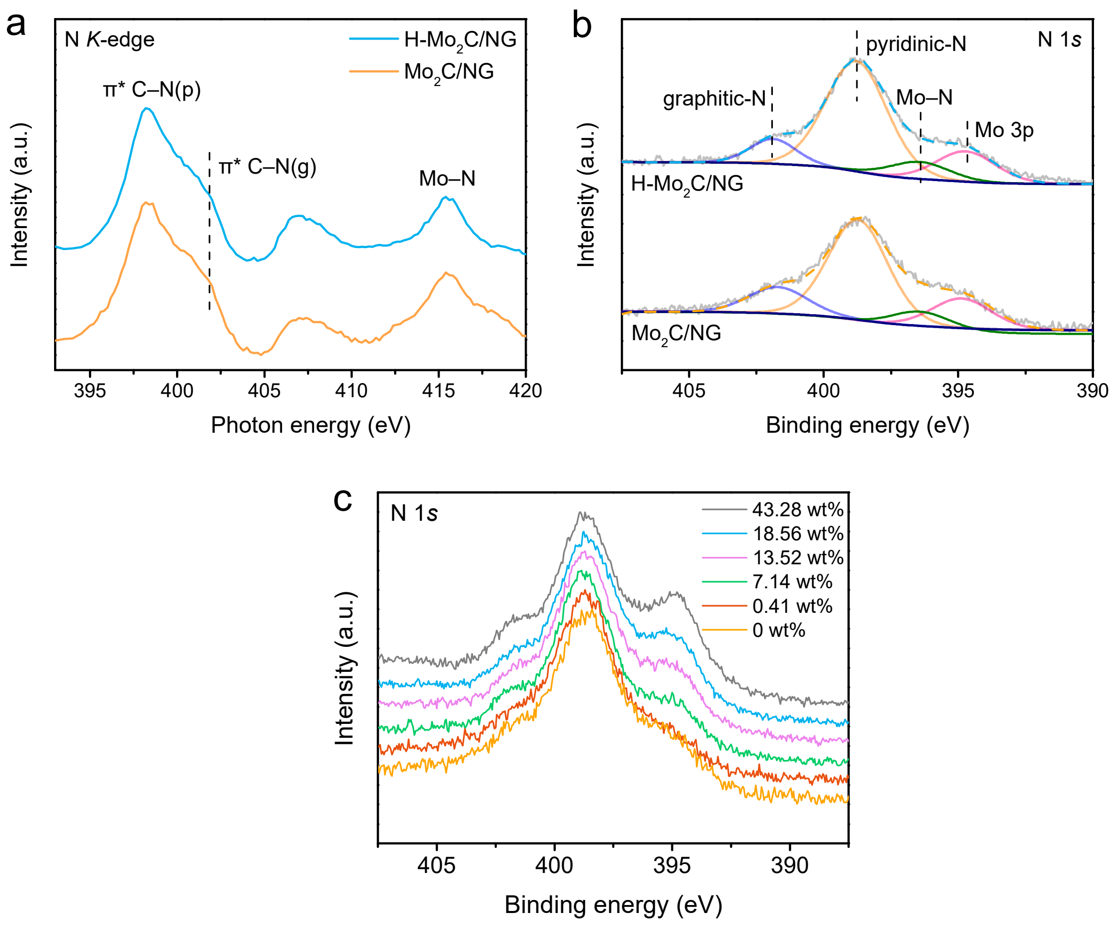

Supplementary Fig. 22. N *K*-edge XANES spectra and N 1*s* XPS spectra. a N *K*-edge XANES spectra of H-Mo_2C/NG and Mo_2C/NG. **b, c** High resolution N 1*s* XPS spectra of Mo_2C hybrids. In the N *K*-edge region, the resonances at 398.3 eV and 401.8 eV are assigned to π* C–N(p) (pyridinic-N) and π* C–N(g) (graphitic-N) in NG[8]. The peak of 407.4 eV is designated as general transition from the N 1*s* core level to the C–N σ* state[9]. In the N 1*s* spectra, the peaks located at 394.7, 396.4, 398.8, and 401.9 eV can be ascribed to Mo 3*p*, Mo–N, pyridinic-N, and graphitic-N species, respectively[6]. The intensity of Mo–N species increases slightly with the increase of water content, indicating that a certain water can also promote N-doping of Mo_2C. Moreover, although the nitrogen concentration in graphene for Mo_2C/NG is higher than that of H-Mo_2C/NG, the types and proportions of nitrogen species in both hybrids are basically the same.

Meanwhile, DFT calculations show that the free energies of hydrogen adsorption on graphitic-N and pyridinic-N are 0.74 and 0.85 eV[5], respectively, which are still much larger than that of Mo_2C (–0.41 eV)[7]. These results indicate that the Mo_2C nanocrystals dominate the HER activity, and the effect of the slight nitrogen concentration difference in graphene on the activity can be entirely ignored.

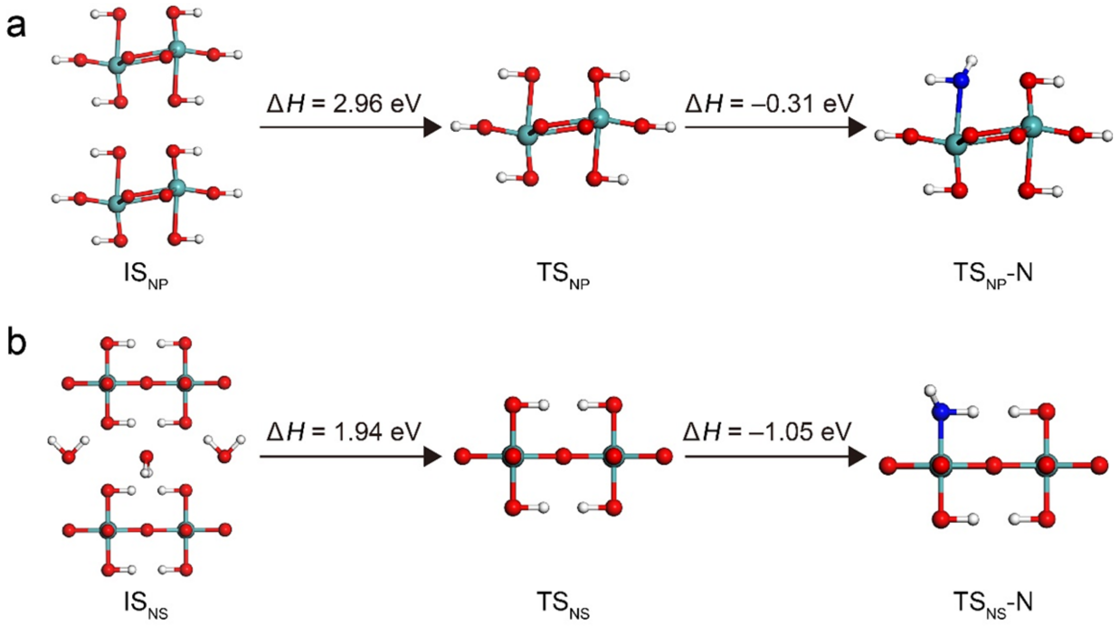

Supplementary Fig. 23. Calculated reaction Δ*H* of ammonia reaction with MoO_3 a without and b with water. The cyan, dark gray, red, blue, and white balls represent Mo, C, O, N, and H atoms, respectively.

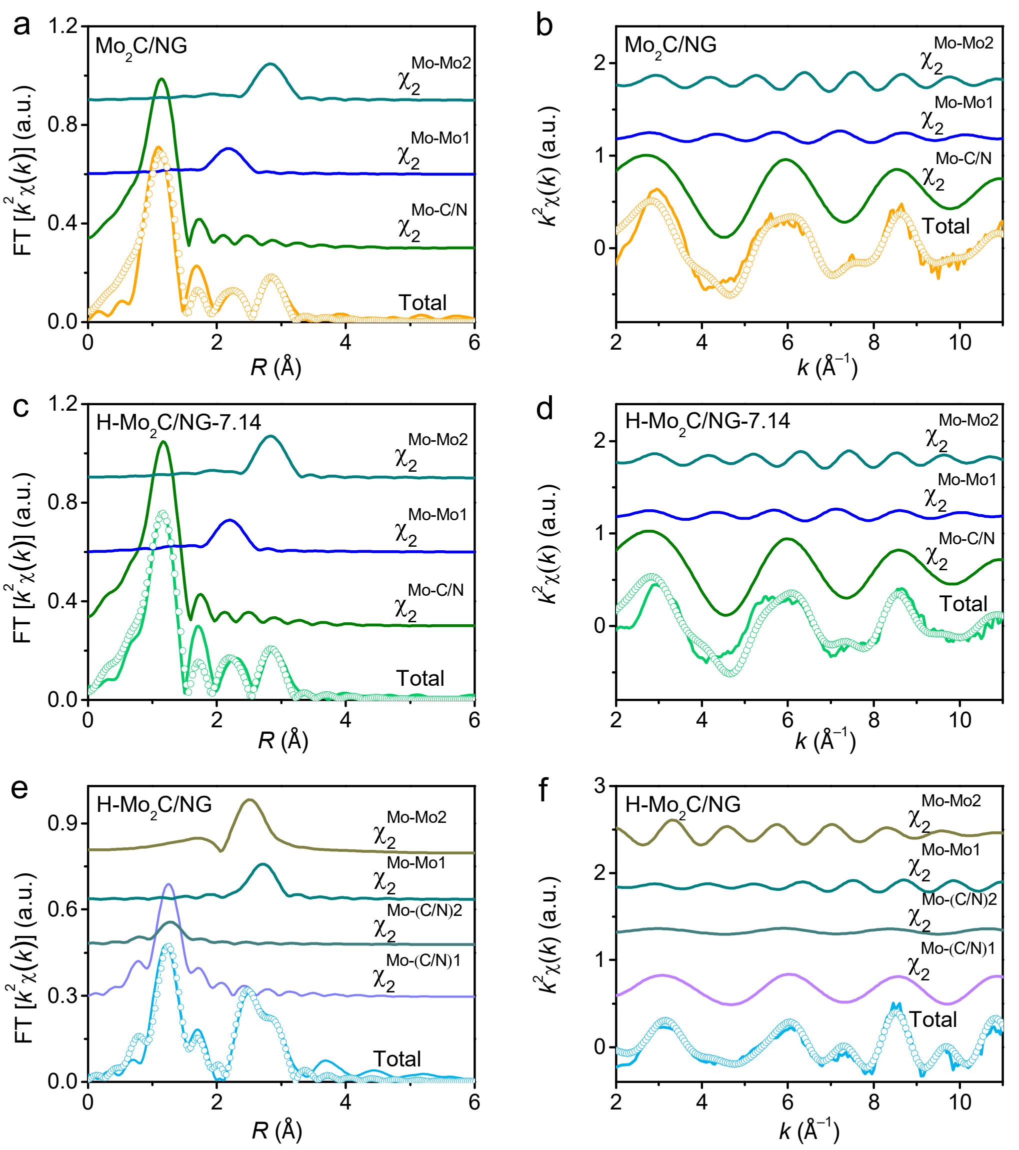

Supplementary Fig. 24. EXAFS fitting curves of Mo_2C hybrids at a, c, e *R*-space and b, d, f *k*-space. a, **b** Mo_2C/NG, **c**, **d** H-Mo_2C/NG-7.14, and **e**, **f** H-Mo_2C/NG. In H-Mo_2C/NG, Mo atoms have two coordination interactions of Mo–(C/N)1 (1.78 Å) and Mo–(C/N)2 (1.86 Å), and the corresponding CN values are estimated to be 1.1 and 1.6, respectively (Supplementary Table 1). While Mo atoms in H-Mo_2C/NG-7.14 and Mo_2C/NG have only one coordination path of Mo–C/N (1.83 and 1.85 Å) with CN of 2.8 and 3.2, respectively. The CN of Mo–C/N in H-Mo_2C/NG is 2.7, which is slightly lower than those of H-Mo_2C/NG-7.14 (CN = 2.8) and Mo_2C/NG (CN = 3.2),

confirming that water induces low coordination Mo atoms in Mo_2C NSs. The CN values of Mo–Mo in H-Mo_2C/NG (CN = 3.8), H-Mo_2C/NG-7.14 (CN = 3.2), and Mo_2C/NG (CN = 3.3) are close, but much lower than that of bulk Mo_2C (CN = 12), which can be attributed to the high-angle GBs in ultrathin Mo_2C NSs and the high dispersion of small-sized Mo_2C NPs[10]. In a typical water-assisted carbonization process, the construction of ultrathin Mo_2C NSs is in carbon-rich environment, however, due to the lattice distortion of Mo_2C nanodomains near GBs, Mo atoms at GBs tend to be coordinately unsaturated.

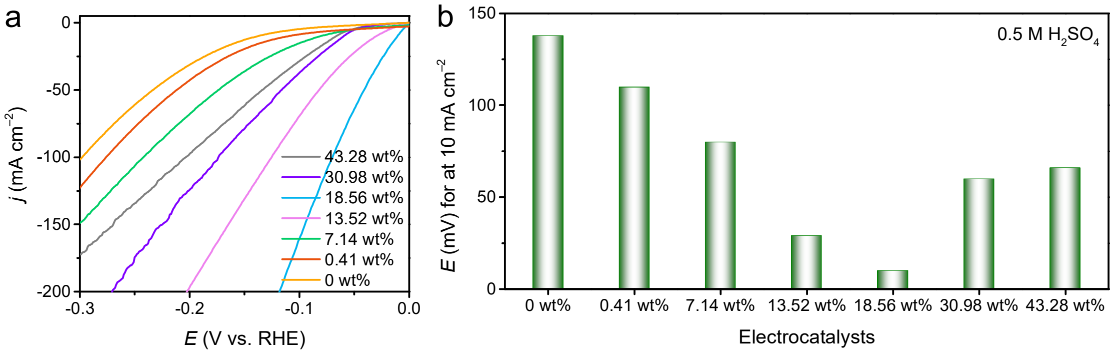

Supplementary Fig. 25. Effects of the water content in MoO_3/RGO on HER activity of Mo_2C hybrids in 0.5 M H_2SO_4. The **a** polarization curves and **b** η_{10}.

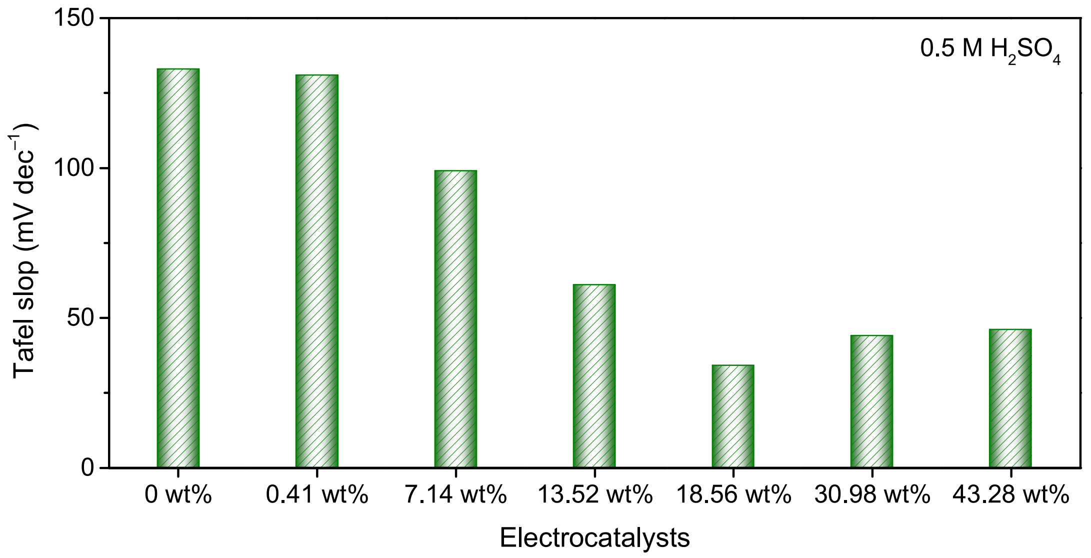

Supplementary Fig. 26. The Tafel slopes of Mo_2C hybrids in 0.5 M H_2SO_4.

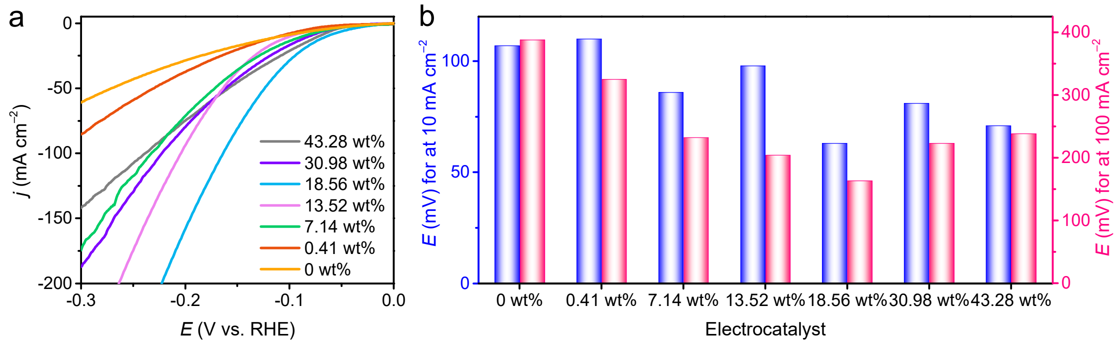

Supplementary Fig. 27. Effects of the water content in MoO_3/RGO on HER activity of Mo_2C hybrids in 1.0 M KOH. The **a** polarization curves and **b** η_{10}.

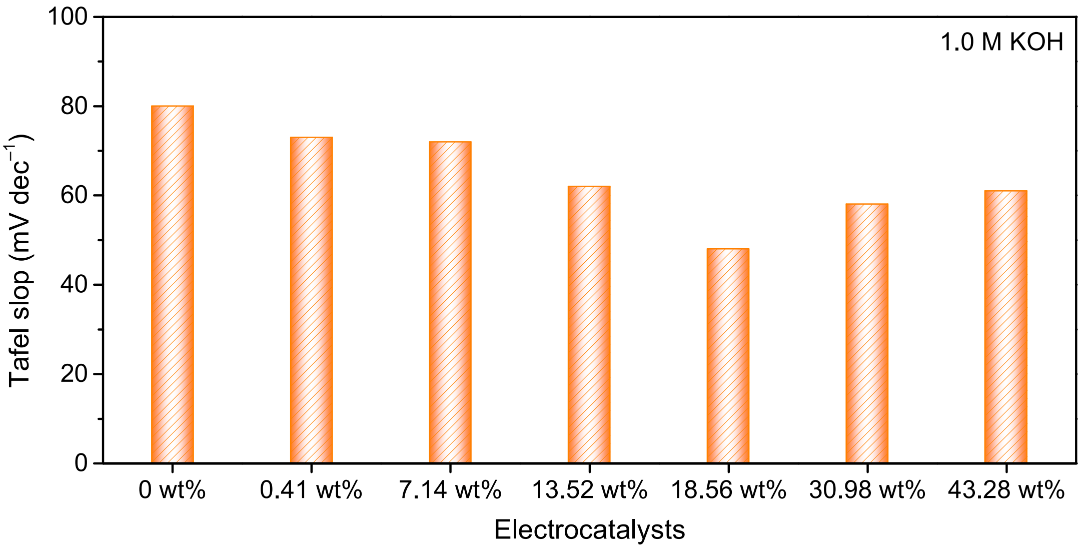

Supplementary Fig. 28. The Tafel slopes of Mo_2C hybrids in 1.0 M KOH.

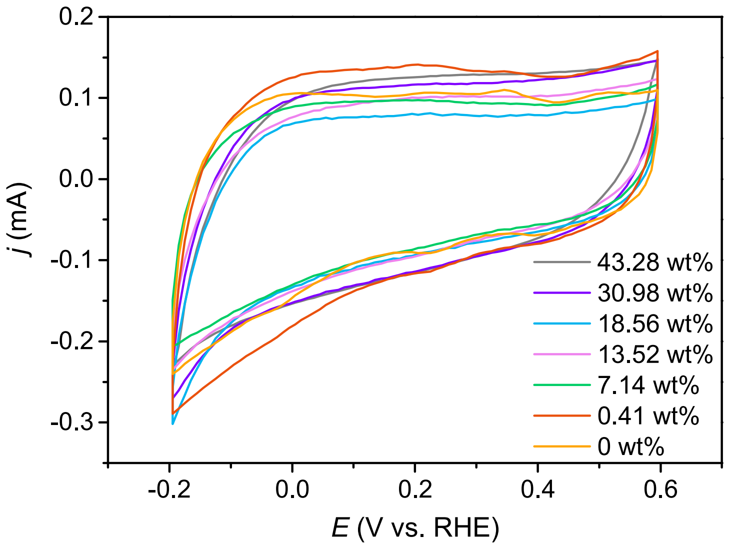

Supplementary Fig. 29. CV cycles of Mo_2C hybrids in PBS (pH 7). The integrated charge of anodic curve for the whole potential range was used to estimate the number of active sites of Mo_2C hybrids.

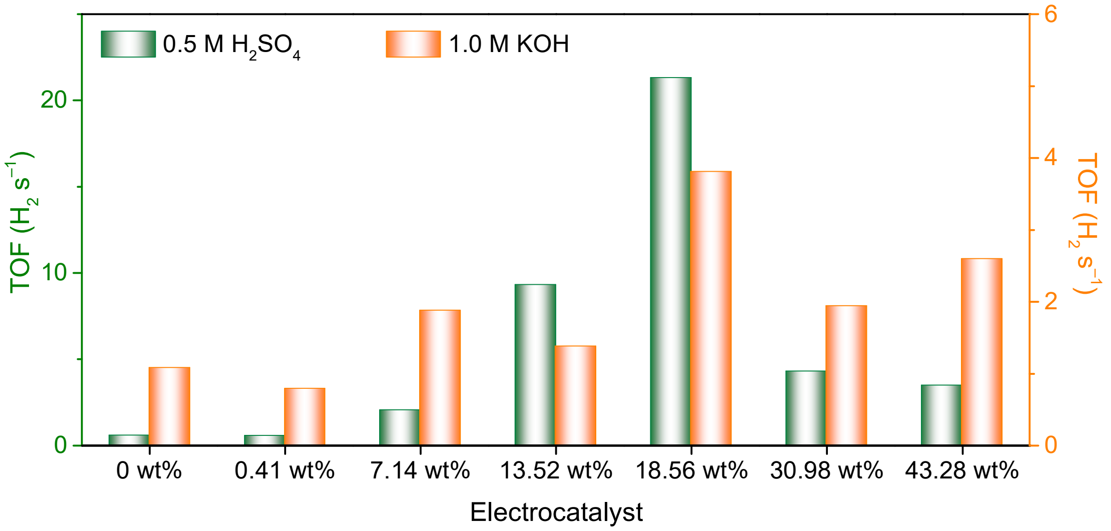

Supplementary Fig. 30. TOF values of Mo_2C hybrids at η = 100 mV in 0.5 M H_2SO_4 and 1.0 M KOH.

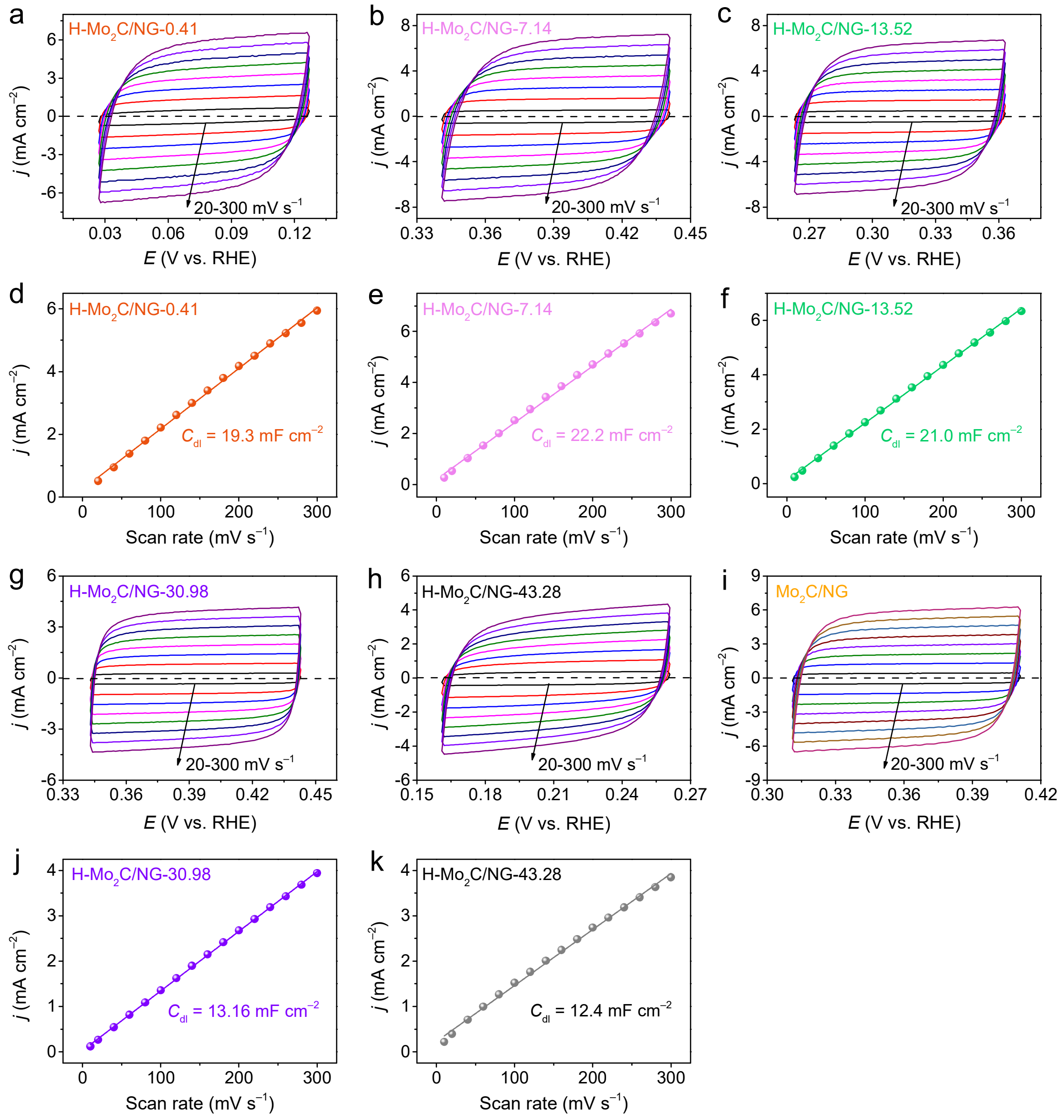

Supplementary Fig. 31. The CV curves with varying scan rates from 20 to 300 mV s^{-1} in 1.0 M KOH and the corresponding capacitive current as a function of scan rate images. a, **d** H-Mo_2C/NG-0.41, **b**, **e** H-Mo_2C/NG-7.14, **c**, **f** H-Mo_2C/NG-13.52, **g**, **j** H-Mo_2C/NG-30.98, **h**, **k** H-Mo_2C/NG-43.28, and **i** Mo_2C/NG.

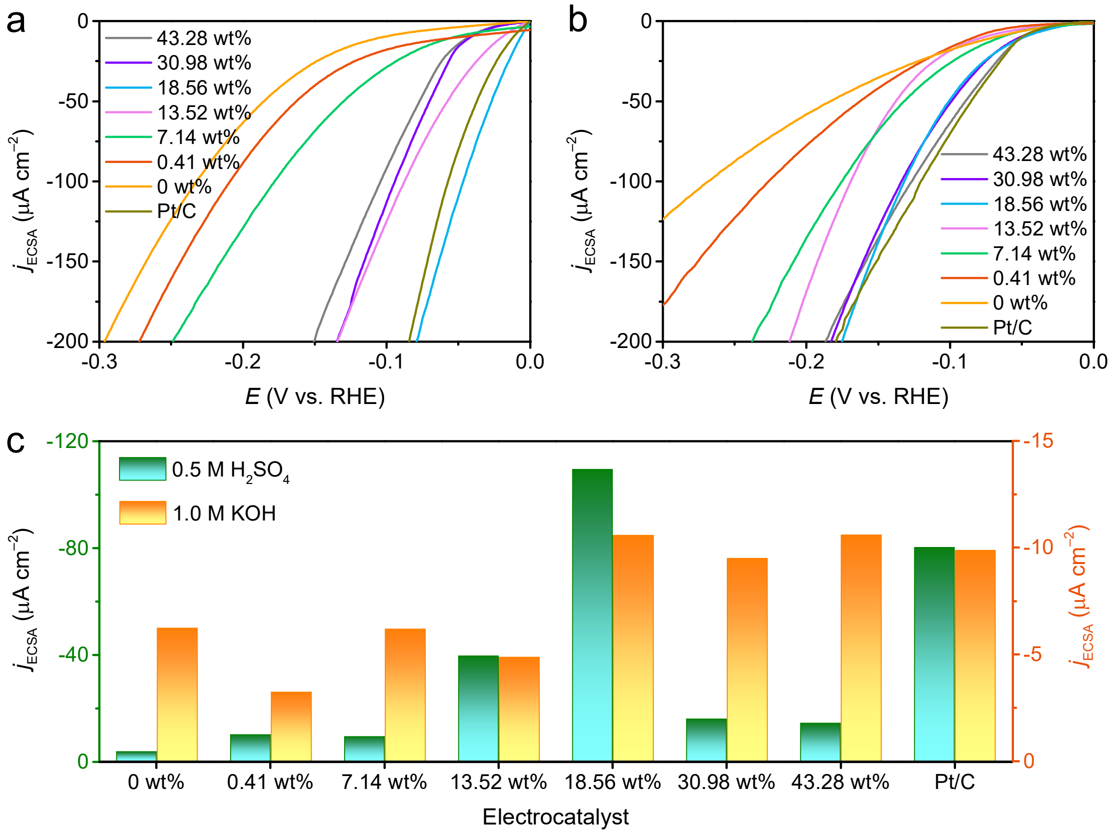

Supplementary Fig. 32. j_{ECSA} of Mo_2C hybrids. a 0.5 M H_2SO_4 and **b** 1.0 M KOH. **c** j_{ECSA} of Mo_2C hybrids at η = 50 mV. The ECSA of Pt/C catalyst is 533.75 cm^2 [11].

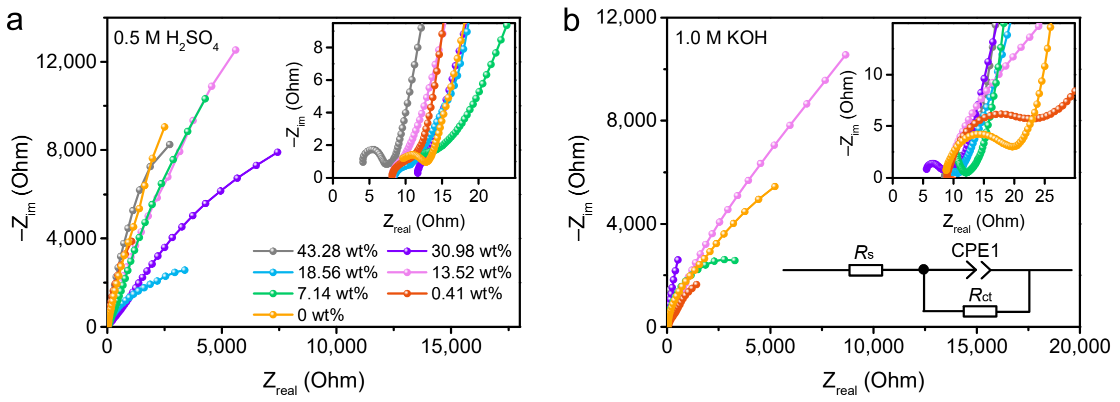

Supplementary Fig. 33. Electrochemical impedance spectroscopy (EIS) analysis. The inset in panel shows the corresponding Nyquist plots at a high-frequency range in **a** 0.5 M H_2SO_4 and **b** 1.0 M KOH. The bottom inset in **b** shows the equivalent circuit for H-Mo_2C/NG electrode. R_s, R_{ct}, and CPE1 represent the resistance of electrolyte solution, charge-transfer resistance, and chemical capacitance, respectively[12].

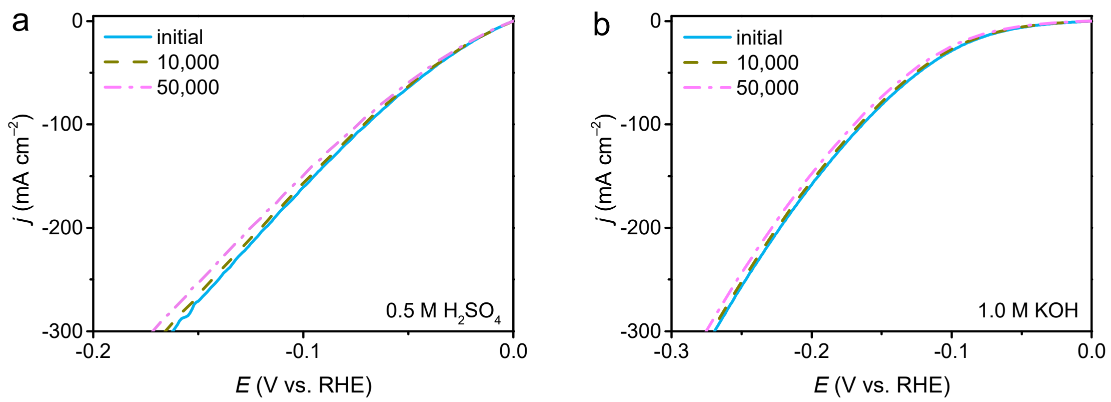

Supplementary Fig. 34. Polarization curves recorded for H-Mo_2C/NG catalyst before and after 10,000 and 50,000 potential cycles in 0.5 M H_2SO_4 and b 1.0 M KOH.

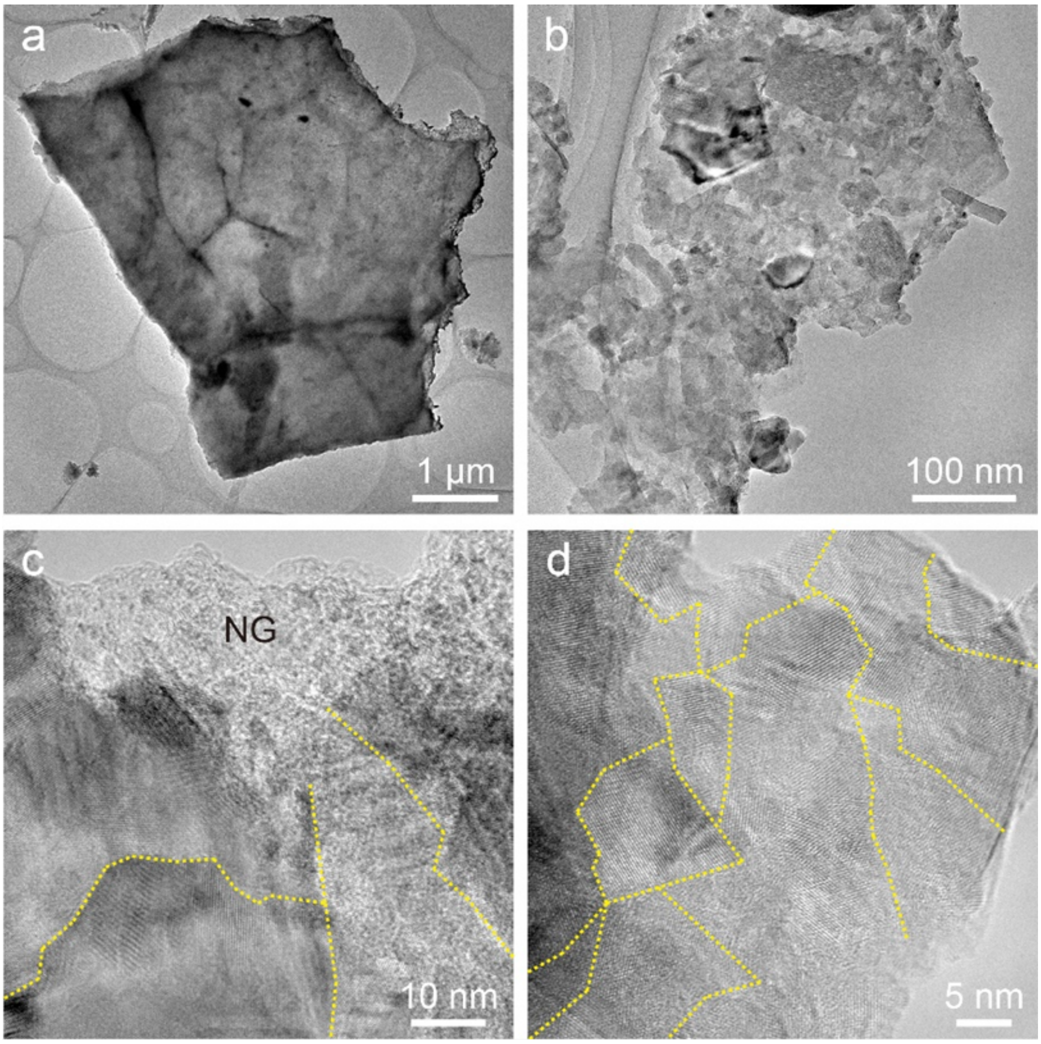

Supplementary Fig. 35. TEM characterization of H-Mo_2C/NG on CFP after cycling test. During the HER process, the H_2 generated at basal-plane sites are trapped in the Mo_2C layers, and then perforates or peels away the Mo_2C layers to escape, resulting in thinner Mo_2C NSs[13].

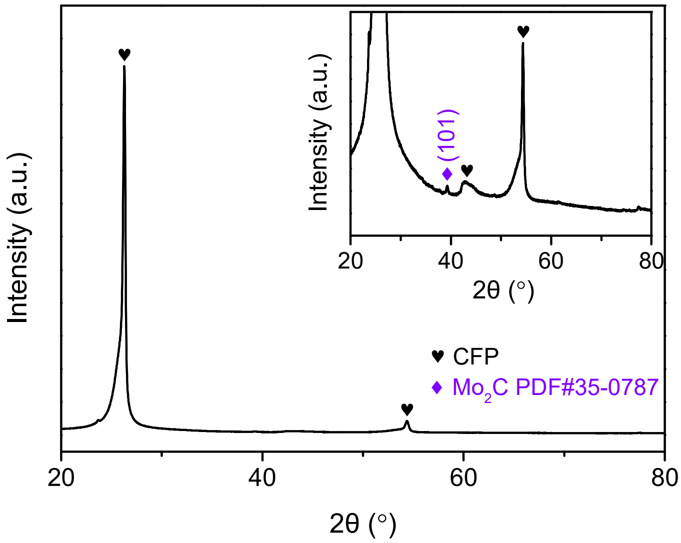

Supplementary Fig. 36. XRD characterization of H-Mo_2C/NG on CFP after cycling test.

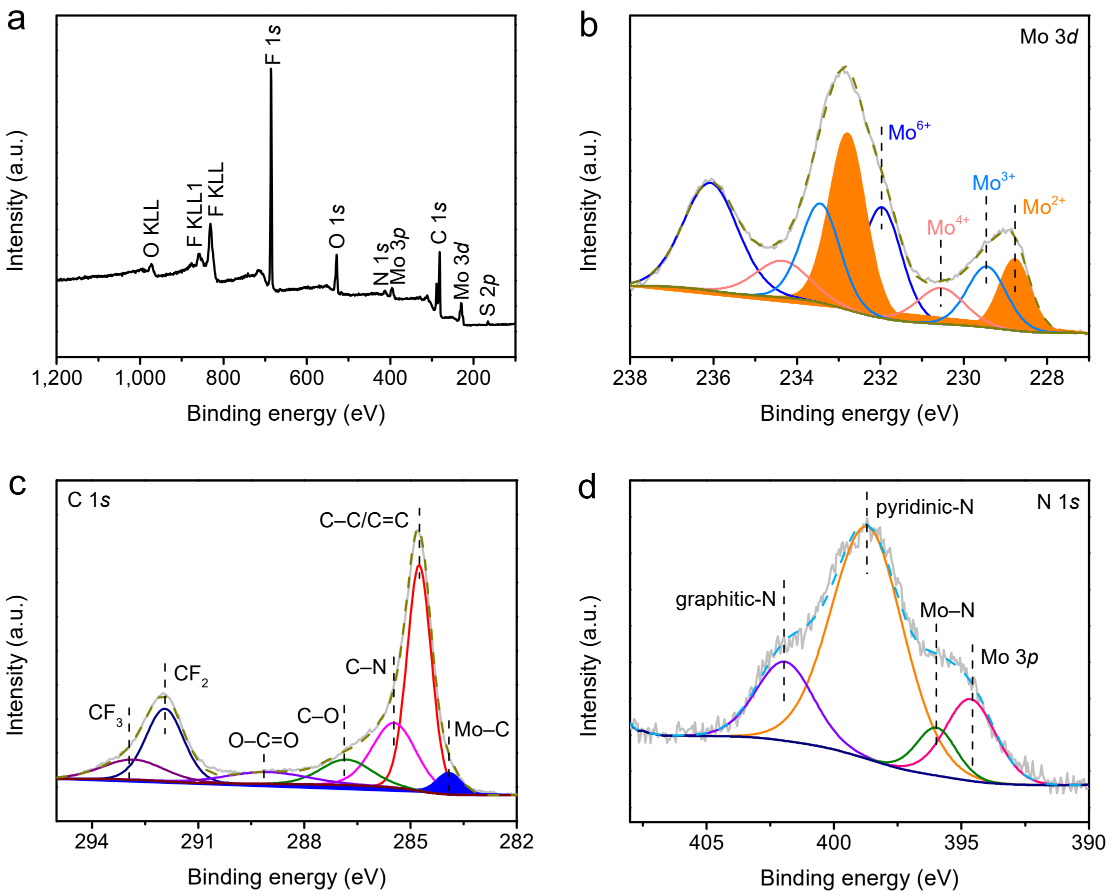

Supplementary Fig. 37. XPS characterization of H-Mo_2C/NG on CFP after cycling test. **a** XPS survey, high resolution XPS spectra of **b** Mo 3*d*, **c** C 1*s*, and **d** N 1*s*. The Mo 3*d* spectrum is deconvoluted into four pairs peaks of Mo^{2+}, Mo^{3+}, Mo^{4+}, and Mo^{6+} species. The Mo^{2+} is from carbides, and Mo^{3+} is assigned as nitrides; the Mo^{4+} and Mo^{6+} can be ascribed to molybdenum oxides species. Deconvolution of the C 1*s* spectrum reveals the presence of Mo–C (283.9 eV), C–C/C=C (284.7 eV), C–N (285.5 eV), and O=C–O (289.2 eV) bonds, as well as the CF_2 (291.9) and CF_3 (292.9 eV) species in Nafion introduced during the electrochemical test[14].

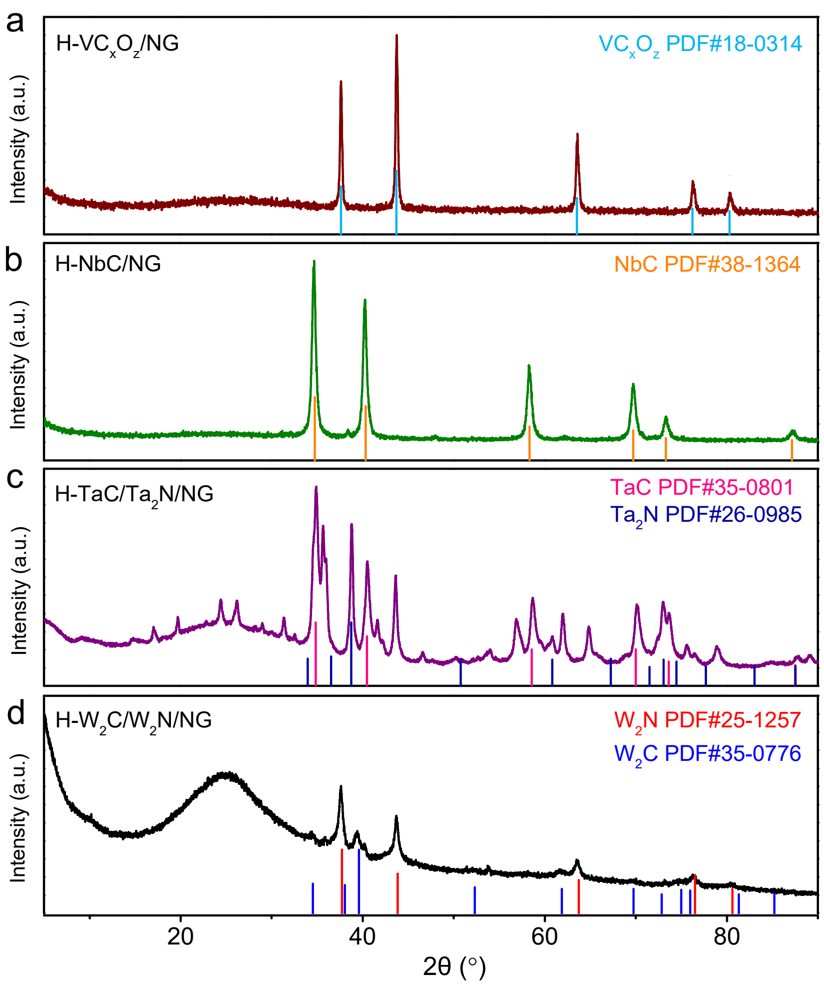

Supplementary Fig. 38. XRD characterization. a H-VC_xO_z/NG, **b** H-NbC/NG, **c** H-TaC/Ta_2N/NG, and **d** H-W_2C/W_2N/NG.

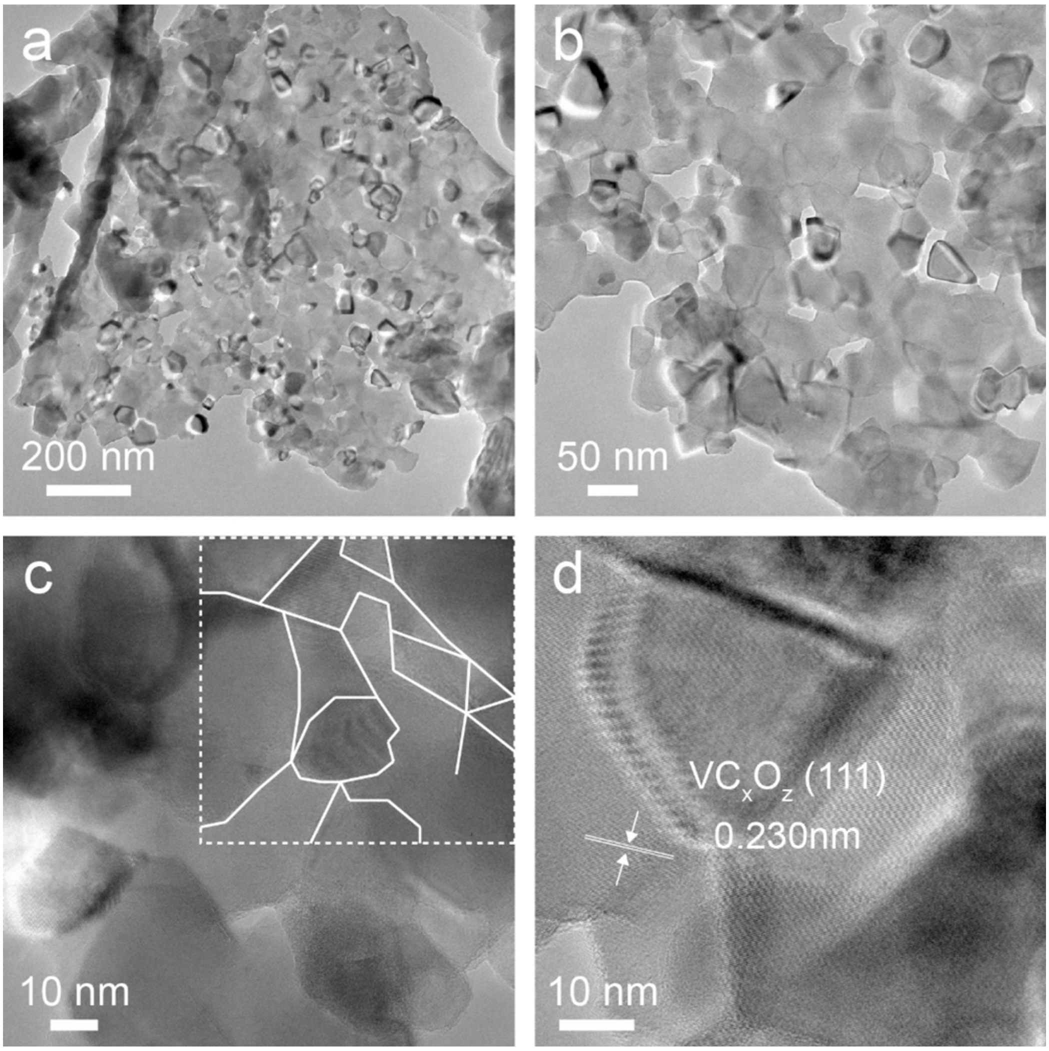

Supplementary Fig. 39. TEM characterization of H-VC_xO_z/NG. The solid lines in boxed region in **c** point out the irregular GBs.

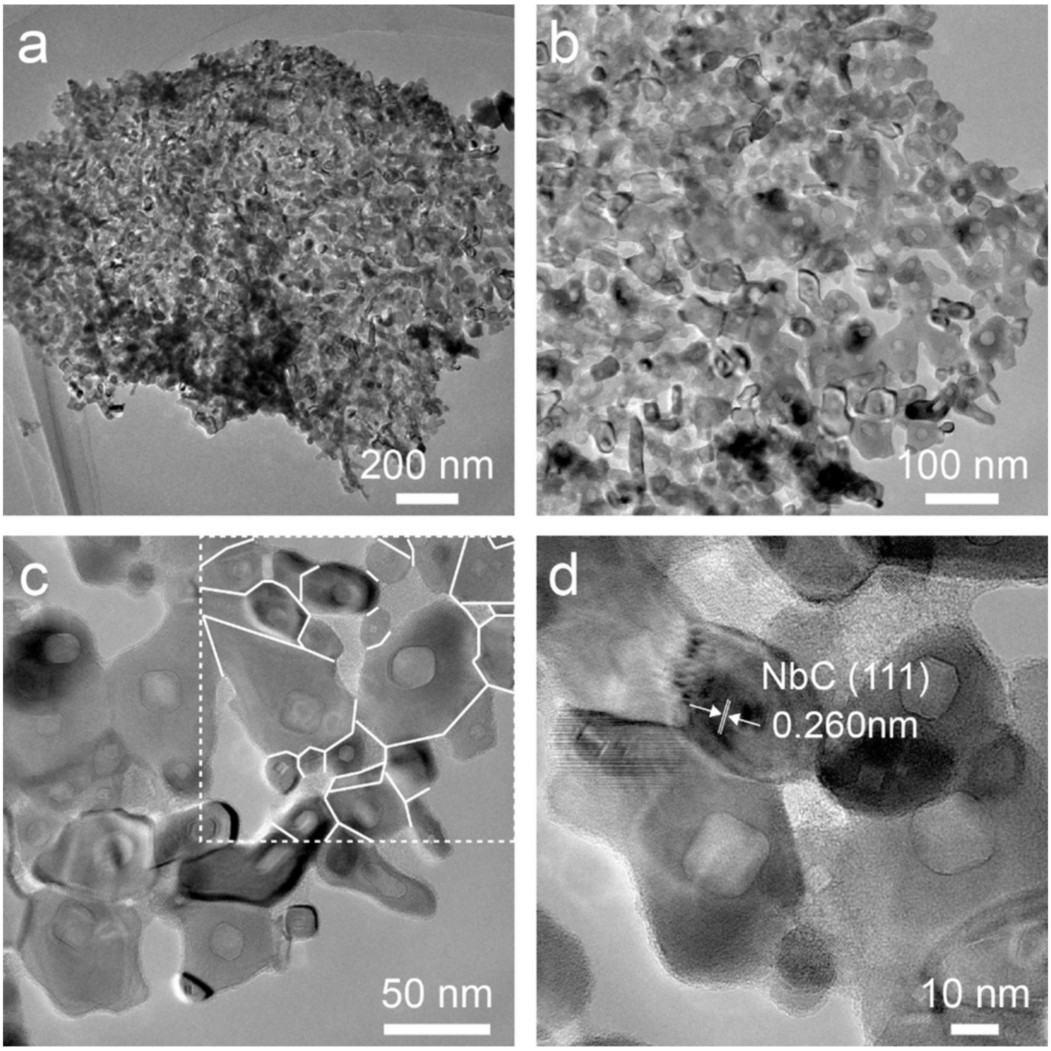

Supplementary Fig. 40. TEM characterization of H-NbC/NG. The solid lines in boxed region in **c** point out the irregular GBs.

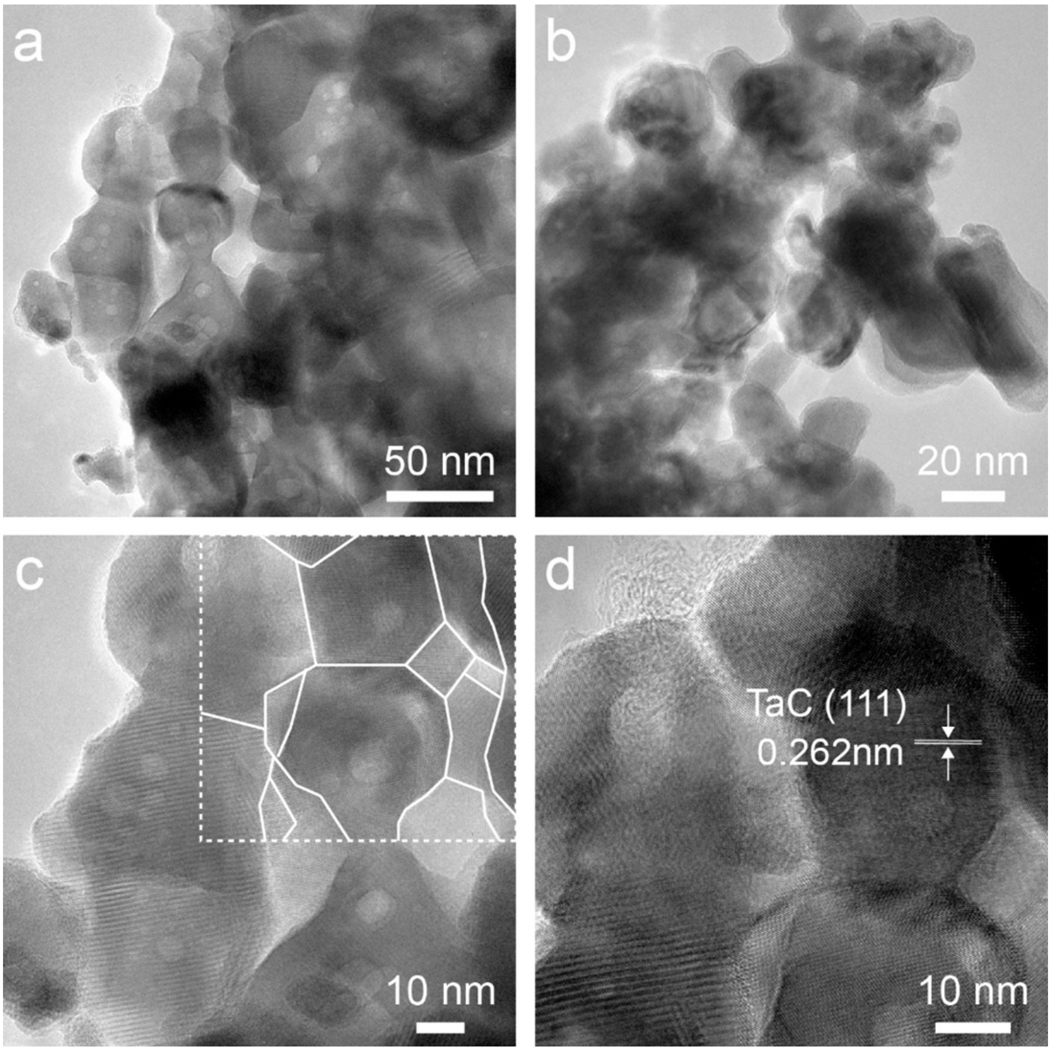

Supplementary Fig. 41. TEM characterization of H-TaC/Ta_2N/NG. The solid lines in boxed region in **c** point out the irregular GBs.

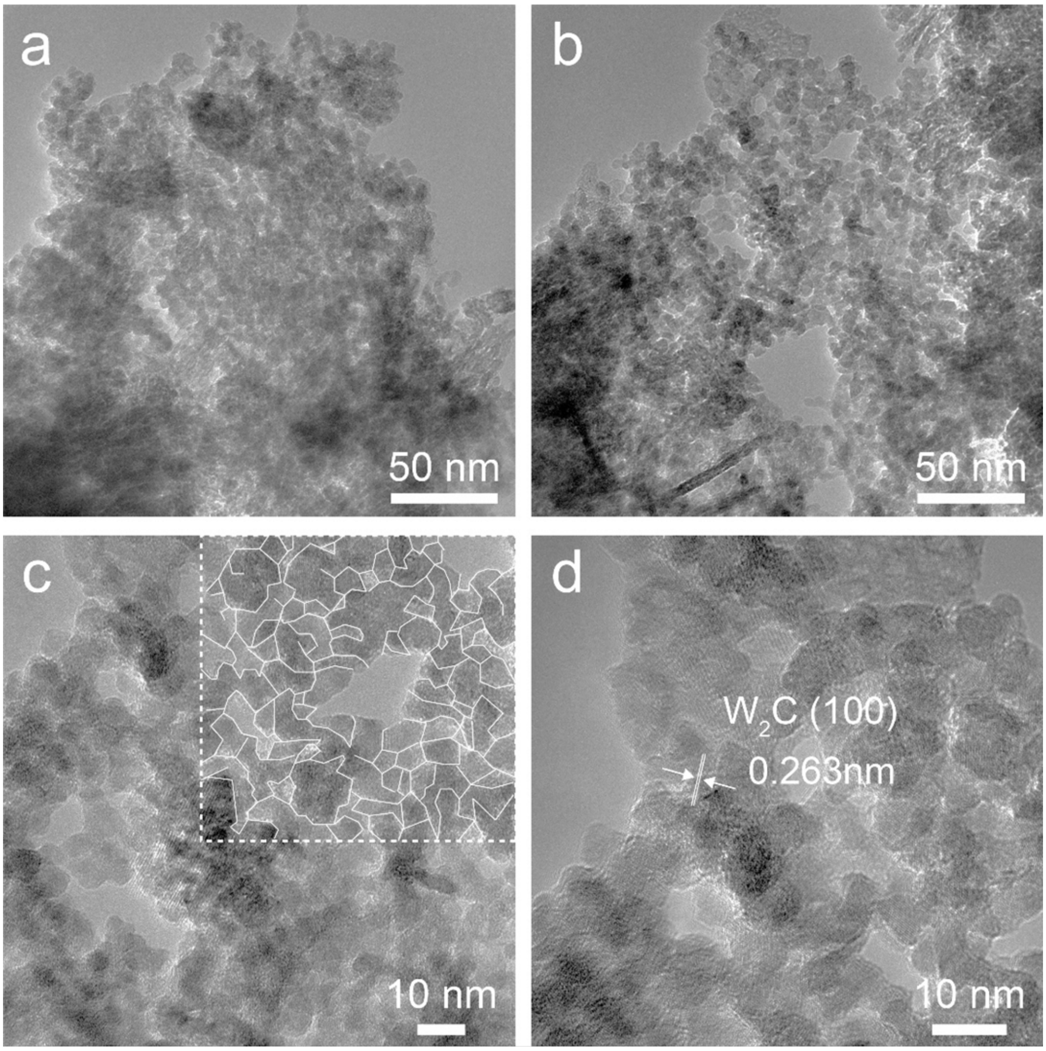

Supplementary Fig. 42. TEM characterization of H-W_2C/W_2N/NG. The solid lines in boxed region in **c** point out the irregular GBs.

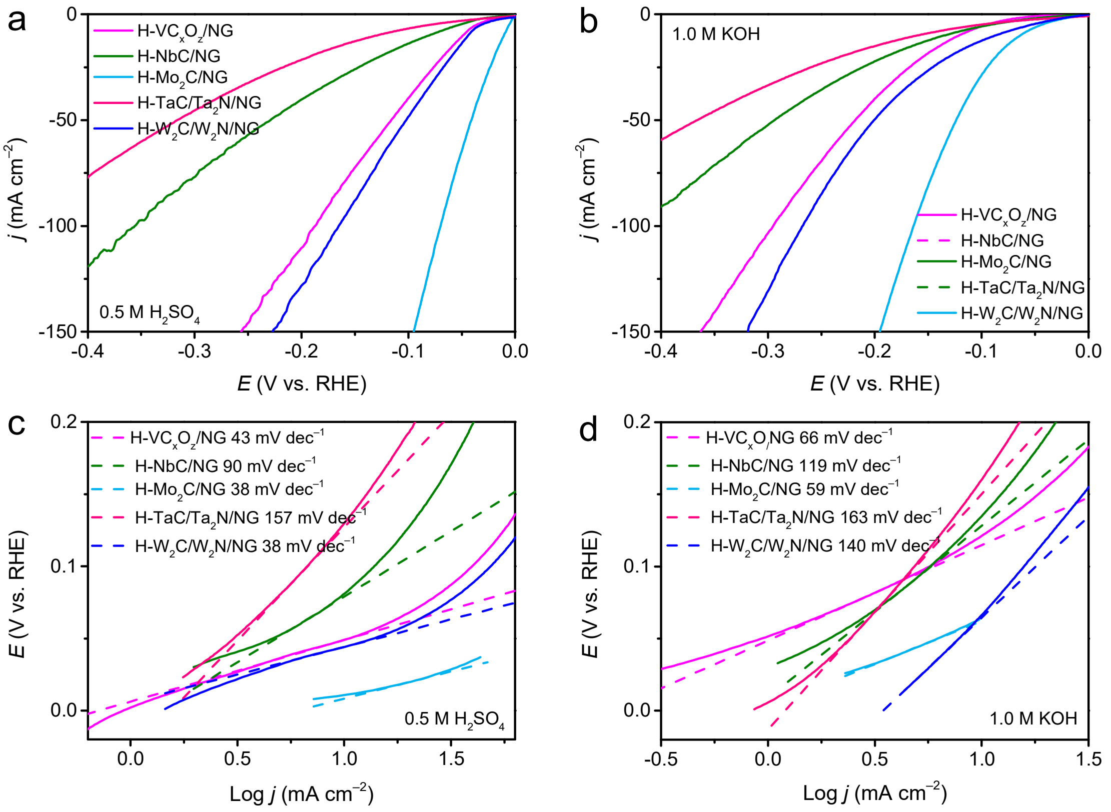

Supplementary Fig. 43. HER performance. a, **b** The polarization curves and the relevant **c**, **d** Tafel plots of H-VC$_x$O$_z$/NG, H-NbC/NG, H-Mo$_2$C/NG, H-TaC/Ta$_2$N/NG, and H-W$_2$C/W$_2$N/NG in **a**, **c** 0.5 M H_2SO_4 and **b**, **d** 1.0 M KOH, respectively.

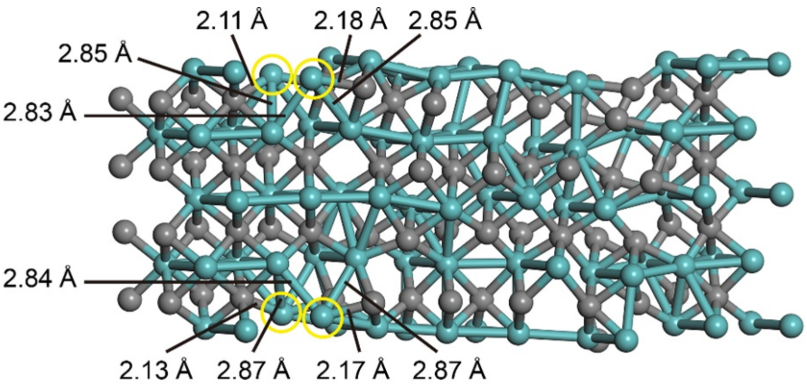

Supplementary Fig. 44. Atomic structure of fcc/hcp GB. Mo atoms with optimized H_{ads} at fcc/hcp GB are highlighted by yellow circles. The cyan and dark gray balls represent Mo and C atoms, respectively. The vicinity of the fcc/hcp GB exhibits optimal H adsorption free energies (such as –0.005, –0.010, –0.013, and 0.058 eV), which are superior to those of the sites far from the GBs (such as –0.324, –0.358, –0.428, and –0.517 eV) (Fig. 6e).

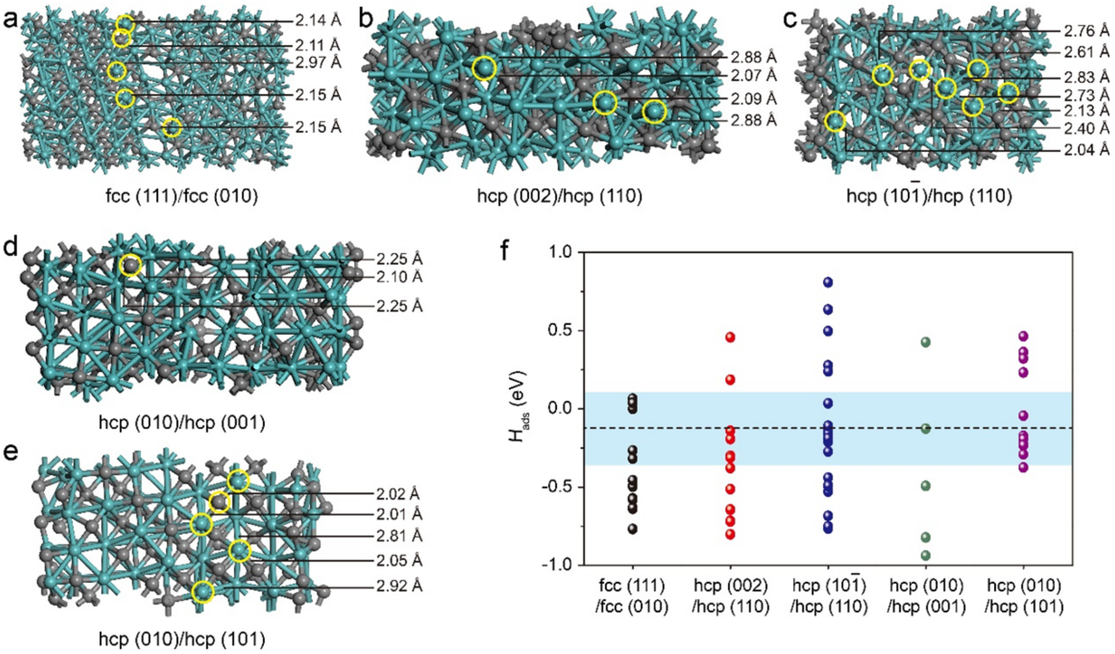

Supplementary Fig. 45. Atomic structure of fcc/fcc and hcp/hcp GBs. a fcc/fcc and **b-e** hcp/hcp GBs. **f** H_{ads} diagram. The dashed line is the H_{ads} of Pt (111) (–0.121 eV). The data points in the shaded area in **f** are generated using the adsorption sites indicated by the yellow circles. The cyan and dark gray balls represent Mo and C atoms, respectively.

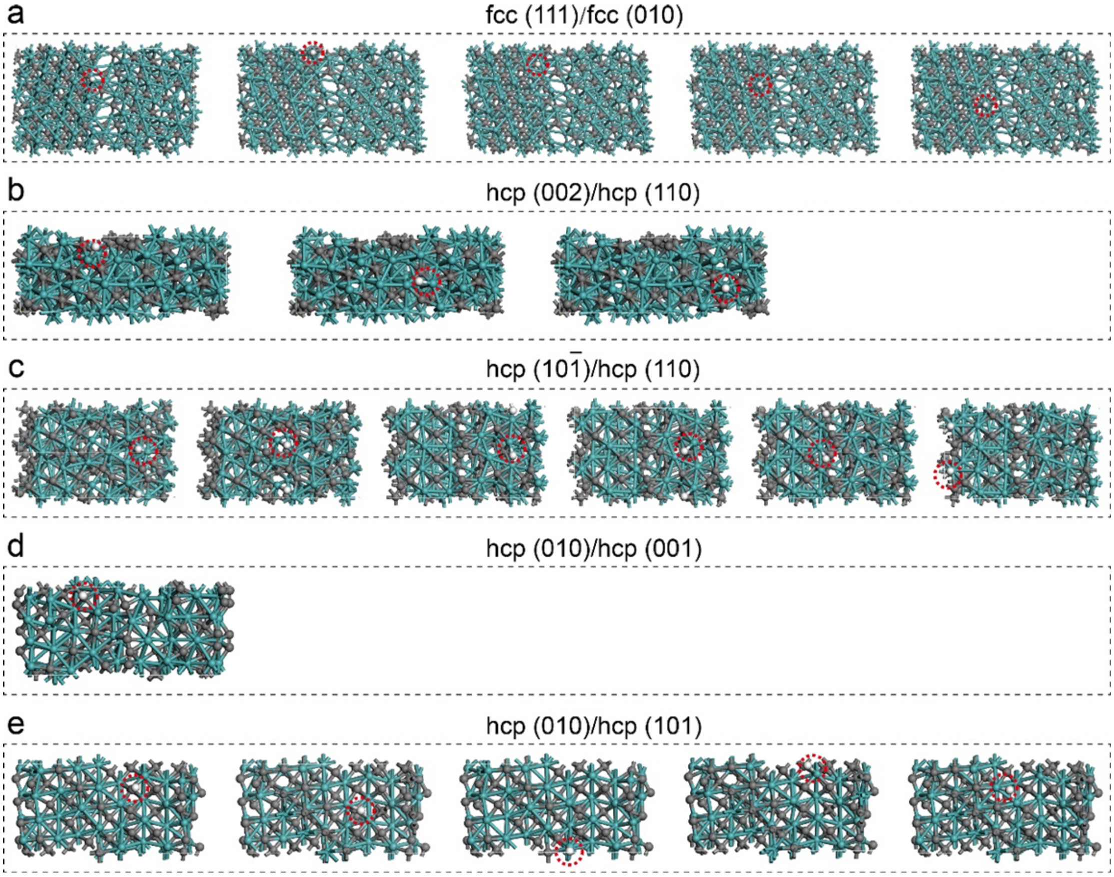

Supplementary Fig. 46. Chemisorption models of the H intermediate in fcc/fcc and hcp/hcp GBs. Mo and C atoms with optimized H_{ads} are highlighted by red dotted circles. The cyan, dark gray, and white balls represent Mo, C, and H atoms, respectively.

Supplementary Tables

Supplementary Table 1. The Mo *K*-edge EXAFS curve fitting parameters[a].

Catalysts	Path	CN	*R* (Å)	σ^2 ($\times10^{-3}$ Å^2)	ΔE_0 (eV)	*R*, %
Mo foil [b]	Mo−Mo1	12	2.85	3.1	0.6	0.01
MoO_3 [c]	Mo−O1	3	1.77	4.0	0.3	0.01
	Mo−O2	3	2.13	6.3		
H-Mo_2C/NG [d]	Mo−(C/N)1	1.1	1.78	4.4	8.5	0.3
	Mo−(C/N)2	1.6	1.86	5.0		
	Mo−Mo1	2.0	2.96	11.4		
	Mo−Mo2	1.8	3.10			
H-Mo_2C/NG-7.14 [d]	Mo−C/N	2.8	1.83	4.7	6.4	0.2
	Mo−Mo1	1.8	2.88	5.3		
	Mo−Mo2	1.4	3.17	9.8		
Mo_2C/NG [d]	Mo−C/N	3.2	1.85	4.3	7.8	0.2
	Mo−Mo1	1.9	2.87	6.3		
	Mo−Mo2	1.4	3.20	10.4		

[a]CN, coordination number; *R*, distance between absorber and backscatter atoms; σ^2, Debye–Waller factor to account for both thermal and structural disorders; ΔE_0, inner potential correction; *R* factor (%) indicates the goodness of the fit. Error bounds (accuracies) that characterize the structural parameters obtained by EXAFS spectroscopy were estimated as CN ± 20%; *R* ± 1%; σ^2 ± 20%; ΔE_0 ± 20%. S_0^2 was fixed to 1.0. Bold numbers indicate fixed CN according to the crystal structure. [b]Fitting range: $3.0 \leq k$ (/Å) ≤ 13.5 and $1.4 \leq R$ (Å) ≤ 3.0. [c]Fitting range: $2.0 \leq k$ (/Å) ≤ 12.5 and $1.0 \leq R$ (Å) ≤ 2.0. [d]Fitting range: $2.8 \leq k$ (/Å) ≤ 11.0 and $1.0 \leq R$ (Å) ≤ 3.4.

Supplementary Table 2. Comparison of j_0[a] of Mo_2C hybrids.

Catalysts	0.5 M H_2SO_4 (mA cm^{-2})	1.0 M KOH (mA cm^{-2})
Mo_2C/NG	0.80	0.31
H-Mo_2C/NG-0.41	1.49	0.32
H-Mo_2C/NG-7.14	1.55	0.62
H-Mo_2C/NG-13.52	3.54	0.63
H-Mo_2C/NG	5.47	0.75
H-Mo_2C/NG-30.98	0.38	0.61
H-Mo_2C/NG-43.28	0.39	0.51
Pt/C	2.83	0.40

[a] j_0 values were calculated from Tafel curves using an extrapolation method.

Supplementary Table 3. Summary of HER performance of reported non-precious metal electrocatalysts in acidic conditions.

Catalysts	η_{10} (mV)	Tafel slope (mV dec^{-1})	Electrolyte	Ref.
p-$Ni_{12}P_5$	27.7	30.88	0.5 M H_2SO_4	11
Co-P@PC-750	33	49	0.5 M H_2SO_4	15
Co_9S_8-MoS_2/NF	250	79.58	0.5 M H_2SO_4	16
Co-Fe-P nanotubes	66	72	0.5 M H_2SO_4	17
B-CoP/CNT	39	50	0.5 M H_2SO_4	18
nano-MoC/C-Ns	126	69	0.5 M H_2SO_4	19
Co/Ni-doped MoP	102	58.1	0.5 M H_2SO_4	20
$(Fe_xNi_{1-x})_2P$ nanosheets	81	53.5	0.5 M H_2SO_4	21
Co/Se-MoS_2-NF	104	67	0.5 M H_2SO_4	22
Ni_2P/Ni@C	149	61.2	0.5 M H_2SO_4	23
Fe/P-CoS_2 PCNW	80	56	0.5 M H_2SO_4	24
Cu@MoS_2	92	50.5	0.5 M H_2SO_4	25
$FePSe_3$/NC	80	53	0.5 M H_2SO_4	26
Mo_2C-MoO_x/CC	60	53	1.0 M $HClO_4$	27
PHA-Mo_2C	93	47.3	0.5 M H_2SO_4	28
Mo_2C@BNC	184	68.3	0.5 M H_2SO_4	29
H-Mo_2C/NG	10	38	0.5 M H_2SO_4	This work

Supplementary Table 4. Summary of HER performance of reported non-precious metal electrocatalysts in alkaline conditions.

Catalysts	η_{10} (mV)	Tafel slope (mV dec^{-1})	Electrolytes	Ref.
Co-P@PC-750	76	50	1.0 M KOH	13
Co_9S_8-MoS_2/NF	110	81.7	1.0 M KOH	16
Co-Fe-P nanotubes	86	66	1.0 M KOH	17
B-CoP/CNT	56	69	0.1 M KOH	18
nano-MoC/C-Ns	92	73	0.1 M KOH	19
$(Fe_xNi_{1-x})_2P$ nanosheets	103	76.6	1.0 M KOH	21
Cu@WC	119	88.9	1.0 M KOH	25
Mo_2C@BNC	145	57.4	1.0 M KOH	29
CoP/NiCoP NTs	133	88	1.0 M KOH	30
$Ni_3(Ni_3\cdot HAHATN)_2$	115	45.6	0.1 M KOH	31
Edge-MoS_2/Co_3S_4@NFs	90.3	61.69	1.0 M KOH	32
Ni_1Co_1-P	169	68	1.0 M KOH	33
$CoMo_2S_4$	55	59.6	1.0 M NaOH	34
MoS_2-Mo_2C	56	64.2	1.0 M KOH	35
N-NiMoS	68	86	1.0 M KOH	36
$NiSe_2$@NC	162	88	1.0 M KOH	37
H-Mo_2C/NG	63	59	1.0 M KOH	This work

Supplementary Table 5. The content of molybdenum in Mo_2C hybrids measured by inductively coupled plasma mass spectrometry (ICP-MS).

Catalysts	Mo (wt%)
Mo_2C/NG	8.28
H-Mo_2C/NG-0.41	7.14
H-Mo_2C/NG-7.14	8.07
H-Mo_2C/NG-13.52	8.07
H-Mo_2C/NG	7.98
H-Mo_2C/NG-43.28	8.03

Supplementary Notes

Supplementary Note 1

To fully understand the growth behavior of Mo_2C, Mo_2C hybrids obtained with various water contents were carefully studied by TEM characterization. With the increase of water content, the structure of Mo_2C evolves from NPs (Mo_2C/NG) (Supplementary Fig. 2) to discontinuous NSs (H-Mo_2C/NG-7.14) (Supplementary Fig. 16), then to continuous NSs with rich GBs (H-Mo_2C/NG) (Supplementary Fig. 17), and finally to layered stacked sheets (H-Mo_2C/NG-43.28) (Supplementary Fig. 18). This result indicates that the morphological structure and chemical distribution of Mo_2C are sensitive to water.

Supplementary Note 2

To investigate the role of NH_3 in the formation of Mo_2C NSs with rich GBs, the same procedure of H-Mo_2C/NG without NH_3 is carried out to obtain molybdenum composites. XRD result shows that Mo-Mo_2C heterostructures are formed when there is no NH_3 during carbothermal reaction (Supplementary Fig. 10), indicating that NH_3 is essential for harvesting pure Mo_2C crystalline phase. The incomplete carbonization of MoO_3 intermediate in the absence of NH_3 possibly results from the sluggish carburetion kinetics induced by the slow pyrolysis process of RGO[38]. When NH_3 is introduced into the carbonization process, the nitrogen atoms from the pyrolysis of NH_3 can be quickly supplied to the reduced molybdenum atoms[39], preventing the formation of metallic molybdenum, resulting in trace nitrogen-doped Mo_2C NSs with dense GBs.

Supplementary Note 3

To investigate the effect of surface functional groups of GO precursor on the GB density, Mo_2C hybrids were prepared using GO with various oxidation levels (Supplementary Fig. 11). Using smooth graphite plates with negligible oxygen-containing functional groups as substrate for Mo_2C growth, the obtained Mo_2C nanocrystals are unevenly distributed and agglomerated significantly (Supplementary Fig. 12). If the GO support has a low oxidation degree, the resulting Mo_2C NSs display a relatively uniform distribution with a small amount of GBs (Supplementary Fig. 13), which can be attributed to limited nucleation sites during carbothermal reaction[40]. The morphology of Mo_2C product supported with excessively oxidized GO (Supplementary Fig. 14) is slightly agglomerated as compared with the uniformly structure of H-Mo_2C/NG synthesized with common GO as substrate (Fig. 2d)[2]. This result can be attributed to the escape of a large number of oxygen-containing functional groups on GO during carbonization process, which severely damaged the skeleton structure, resulting in inhibiting the exposure of GBs[41]. Thus, the above results indicate that sufficient oxygen-containing functional groups on GO surface are necessary for the formation of high-density GBs in Mo_2C NSs.

Supplementary Note 4

Calculation of TOF. The TOF is defined as follows:

$$\text{TOF} = \frac{j \times A}{2Fm} \quad (1)$$

where j is the current density (A cm^{-2} = C s^{-1} cm^{-2}) at a specific overpotential (–0.2 ~ 0.6 V vs. RHE), A is the surface area of electrode (cm^2), the factor 1/2 is related to the number of electrons required to generate one molecule of H_2, F is Faraday constant (96485.4 C mol^{-1}) and m is the number of surfaced active sites (moles) those take part in electrochemical HER.

$$m = \frac{Q_A}{2F} \quad (2)$$

Here, the anodic charge (Q_A, Coulombs) was calculated from the anodic part of CV curves measured in PBS (pH 7) at 50 mV s^{-1} as showed in Supplementary Fig. 29.

Supplementary Note 5

Calculation of ECSA. ECSA was estimated by using C_{dl} that was obtained from the linear slope of the plot between scan rate and current density measured by cyclic voltammetry (CV) curves in 1.0 M KOH. The specific capacitance for a flat surface is generally found to be in the range of 20 ~ 60 μF cm^{-2}. Assume that the specific capacitance of a flat surface is 40 μF cm^{-2} for 1 cm^2 of real surface area. ECSA was calculated by the following equation:

$$\mathrm{ECSA} = \frac{\text{Specific capacitance } (\mathrm{mF\ cm^{-2}})}{40\ \mu\mathrm{F\ cm^{-2}\ per\ cm^2}} \quad (3)$$

Supplementary Note 6

DFT calculations. The first-principles calculations based on the DFT were performed within generalized gradient approximation (GGA). Core electron states were represented by the projector augmented-wave method as implemented in the Vienna ab initio simulation package (VASP). The Perdew–Burke–Ernzerhof exchange correlation functional and a plane wave representation for the wave function with a cut-off energy of 450 eV were used. During the simulation lattice parameters were fully relaxed, geometry of crystal was fully optimized on the basis of the criteria of a maximum atomic force of 0.01 eV $Å^{-1}$ and energy convergence criteria of 0.1 meV before the electron structure and total energy calculation. The VASPSOL[42] function was employed to mimic the solvation environment. 15 Å vacuum space and dipole correction were added to the 2D slab model to remove the convergence problem. Different supercells (2×2×1 to 4×4×1) were adopted during the calculation and the *k*-mesh point convergence test was conducted for each supercell. The strength of H atom binding to the surface was determined by the bond strength of the H–Mo bond, which was determined by the orbital overlap between the H and surface Mo atoms. The classical molecular orbital overlap theory was used to explain the overlap between the d_z^2 orbital of Mo and *s* orbital of H. Moreover, the detailed electronic structures of Mo_2C hybrids were demonstrated by PDOS with the DFT calculation.

The free energy of the H adsorption was calculated as:

$$H_{ads} = E(H^*) + ZPE(H^*) - TS(H^*) - E(^*) - 1/2[E(H_2) + ZPE(H_2) - TS(H_2)] \quad (4)$$

Where the star (*) indicate the substrate, H* means the adsorbed state.

In 1958, Parson first pointed out that a maximum exchange current density would be attained when the hydrogen adsorption free energy was close to thermoneutral (~ 0 eV)[43]. Norskov et al. plotted hydrogen adsorption free energy from DFT calculations versus experimental HER exchange current density[44], and observed a volcano-shaped variation with the peak position close to platinum–note that platinum was the state-of-the-art HER catalyst with an almost zero overpotential and small Tafel slope. This volcano plot was further consummated in other studies in the literatures[45, 46]. These reports suggest that hydrogen adsorption free energy can be exploited as an effective descriptor in the design, engineering and optimization of HER catalysts. In our case, the H is adsorbed on top of the Mo atoms, which is the same as that on the surface of Pt. Therefore, the computational hydrogen electrode (CHE) method was used in this work to calculate hydrogen adsorption free energy. In addition, dilution models consistent with experiment were employed at very small applied potential.

According to the HAADF-STEM images of Mo_2C NSs, the computational models of Mo_2C with fcc/hcp, fcc/fcc, and hcp/hcp GBs were constructed, respectively. Specifically, Fig. 3e shows the superstructure of fcc/hcp heterophase GB in Mo_2C nanosheets. Along the fcc/hcp heterophase edges, both hcp phase with "ABAB" stacking and fcc phase with "ABCABC" stacking are observed along the close-packed $[110]_f/[11\bar{2}0]_h$ direction, which clearly reveals that the heterophase Mo_2C GB is generated through the chemical connection of fcc(111) and hcp(002) slabs. By analogy, we used the software of VESTA to successfully construct fcc(111)/fcc(010) (Supplementary Fig. 19), hcp(002)/hcp(110), hcp(10$\bar{1}$)/hcp(110), hcp(010)/hcp(001),

and hcp(010)/hcp(101) models (Fig. 3a, c and Supplementary Fig. 16) to simulate the fcc/hcp, fcc/fcc, and hcp/hcp Mo_2C GBs in actual material. The structural models obtained by subsequent optimization screening were shown in Supplementary Figs. 44, 45.

The reaction enthalpy during Mo_2C synthesis was calculated by the enthalpy difference between the products and reactants after geometry optimization. MoO_3/RGO intermediates with different water contents (0, 0.41, 7.14, 13.52, 18.56, 30.98, and 43.28 wt%) were obtained by freeze-drying. And HER performance tests found that Mo_2C/NG and H-Mo_2C/NG obtained from anhydrous MoO_3 and MoO_3 with water content of 18.56 wt% had the worst and best activities, respectively (Fig. 5). Accordingly, we choose the MoO_3 structure model with water free and water content of 18.56 wt% to study the mechanism of water action in the carbothermal reaction. We searched MoO_3 structures with different water contents in the previously published literatures, and found that the structure of IS_{NS} with water content of 16% was similar to our experimental synthesized structure, and defined anhydrous MoO_3 as the IS_{NP} structure. Since MoO_3 is a typical layered structure, the layers are staggered and arranged by van der Waals force, which makes it prone to decompose at high temperature. In our case, IS_{NP} and IS_{NS} first absorb heat and dissociate into transition states of TS_{NP} and TS_{NS}, respectively, and they then react with C atoms in RGO to form Mo_2C.

The reaction enthalpy was calculated as:

$$\Delta H = H_{\text{products}} - H_{\text{reactants}} \quad (5)$$

Supplementary References

1. Chen, W. F. et al. Highly active and durable nanostructured molybdenum carbide electrocatalysts for hydrogen production. *Energy Environ. Sci.* **6**, 943–951 (2013).

2. Shuai, S. et al. Improved synthesis of graphene oxide with controlled oxidation degree by using different dihydrogen phosphate as intercalators. *Chem. Phys.* **539**, 110938 (2020).

3. Lavin-Lopez, M. d. P., Romero, A., Garrido, J., Sanchez-Silva, L. & Valverde, J. L. Influence of different improved hummers method modifications on the characteristics of graphite oxide in order to make a more easily scalable method. *Ind. Eng. Chem. Res.* **55**, 12836–12847 (2016).

4. Li, S. et al. Metal-organic precursor-derived mesoporous carbon spheres with homogeneously distributed molybdenum carbide/nitride nanoparticles for efficient hydrogen evolution in alkaline media. *Adv. Funct. Mater.* **29**, 1807419 (2019).

5. Wang, Z. et al. Theoretical calculation guided electrocatalysts design: nitrogen saturated porous Mo_2C nanostructures for hydrogen production. *Appl. Catal. B: Environ.* **257**, 117891(2019).

6. Yan, H. et al. Holey reduced graphene oxide coupled with an Mo_2N-Mo_2C heterojunction for efficient hydrogen evolution. *Adv. Mater.* **30**, 1704156 (2018).

7. Guo, Y. et al. Assembly of hollow mesoporous nanoarchitectures composed of ultrafine Mo_2C nanoparticles on N-doped carbon nanosheets for efficient

electrocatalytic reduction of oxygen. *Mater. Horiz.* **4**, 1171–1177 (2017).

8. Zheng, Y. et al. High electrocatalytic hydrogen evolution activity of an anomalous ruthenium catalyst. *J. Am. Chem. Soc.* **138**, 16174−16181 (2016).

9. Zhang, L. S., Liang, X. Q., Song, W. G. & Wu, Z. Y. Identification of the nitrogen species on N-doped graphene layers and Pt/NG composite catalyst for direct methanol fuel cell. *Phys. Chem. Chem. Phys.* **12**, 12055–12059 (2010).

10. Shou, H., Ferrari, D., Barton, D. G., Jones, C. W. & Davis, R. J. Influence of passivation on the reactivity of unpromoted and Rb-promoted Mo_2C nanoparticles for CO hydrogenation. *ACS Catal.* **2**, 1408–1416 (2012).

11. Duan, J., Chen, S., Ortíz-Ledón, C. A., Jaroniec, M. & Qiao, S. Z. Phosphorus vacancies that boost electrocatalytic hydrogen evolution by two orders of magnitude. *Angew. Chem. Int. Ed.* **59**, 8181–8186 (2020).

12. Zang, Y. et al. Tuning orbital orientation endows molybdenum disulfide with exceptional alkaline hydrogen evolution capability. *Nat. Commun.* **10**, 1217 (2019).

13. Liu, Y. et al. Self-optimizing, highly surface-active layered metal dichalcogenide catalysts for hydrogen evolution. *Nat. Energy* **2**, 17127 (2017).

14. Jung, J.-H., Jeon, J.-H., Sridhar, V. & Oh, I.-K. Electro-active graphene-Nafion actuators. *Carbon* **49**, 1279–1289 (2011).

15. Wu, J. et al. An efficient cobalt phosphide electrocatalyst derived from cobalt phosphonate complex for all-pH hydrogen evolution reaction and overall water splitting in alkaline solution. *Small* **16**, e1900550 (2020).

16. Kim, M. et al. Covalent 0D–2D heterostructuring of Co_9S_8-MoS_2 for enhanced hydrogen evolution in all pH electrolytes. *Adv. Funct. Mater.* **30**, 2002536 (2020).

17. Chen, J. et al. Co-Fe-P nanotubes electrocatalysts derived from metal-organic frameworks for efficient hydrogen evolution reaction under wide pH range. *Nano Energy* **56**, 225–233 (2019).

18. Cao, E. et al. Boron-induced electronic-structure reformation of CoP nanoparticles drives enhanced pH-universal hydrogen evolution. *Angew. Chem. Int. Ed.* **59**, 4154–4160 (2020).

19. Gao, B. et al. MoC nanodots toward efficient electrocatalytic hydrogen evolution: an interlayer-confined strategy with 2D-zeolite precursor. *J. Mater. Chem. A* **9**, 4724–4733 (2021).

20. Xiao, W. et al. Hierarchical ultrathin carbon encapsulating transition metal doped MoP electrocatalysts for efficient and pH-universal hydrogen evolution reaction. *Nano Energy* **70**, 104445 (2020).

21. Zhang, W. et al. Single-crystalline $(Fe_xNi_{1-x})_2P$ nanosheets with dominant {011 $\bar{1}\bar{1}$} facets: efficient electrocatalysts for hydrogen evolution reaction at all pH values. *Nano Energy* **56**, 813–822 (2019).

22. Zheng, Z. et al. Boosting hydrogen evolution on MoS_2 via co-confining selenium in surface and cobalt in inner layer. *Nat. Commun.* **11**, 3315 (2020).

23. Liu, X. et al. Two birds with one stone: metal-organic framework derived micro-/nanostructured Ni_2P/Ni hybrids embedded in porous carbon for electrocatalysis

and energy storage. *Adv. Funct. Mater.* **29**, 1901510 (2019).

24. Zhang, Y.-Y. et al. Fe/P dual doping boosts the activity and durability of CoS_2 polycrystalline nanowires for hydrogen evolution. *J. Mater. Chem. A* **7**, 5195–5200 (2019).

25. Yao, M. et al. Rational design of self-supported Cu@WC core-shell mesoporous nanowires for pH-universal hydrogen evolution reaction. *Appl. Catal. B: Environ.* **280**, 119451 (2021).

26. Yu, J. et al. Metallic $FePSe_3$ nanoparticles anchored on N-doped carbon framework for all-pH hydrogen evolution reaction. *Nano Energy* **57**, 222–229 (2019).

27. He, L. et al. Molybdenum carbide-oxide heterostructures: in situ surface reconfiguration toward efficient electrocatalytic hydrogen evolution. *Angew. Chem. Int. Ed.* **59**, 3544–3548 (2020).

28. Zhao, L. et al. Active facet regulation of highly aligned molybdenum carbide porous octahedrons via crystal engineering for hydrogen evolution reaction. *Nano Energy* **77**, 105056 (2020).

29. Wu, S. et al. Molybdenum carbide nanoparticles assembling in diverse heteroatoms doped carbon matrix as efficient hydrogen evolution electrocatalysts in acidic and alkaline medium. *Carbon* **171**, 385–394 (2021).

30. Lin, Y. et al. Construction of CoP/NiCoP nanotadpoles heterojunction interface for wide pH hydrogen evolution electrocatalysis and supercapacitor. *Adv. Energy Mater.* **9**, 1901213 (2019).

31. Huang, H. et al. Conductive metal-organic frameworks with extra metallic sites as an efficient electrocatalyst for the hydrogen evolution reaction. *Adv. Sci.* **7**, 2000012 (2020).

32. Peng, O. et al. Hierarchical heterostructured nickle foam-supported Co_3S_4 nanorod arrays embellished with edge-exposed MoS_2 nanoflakes for enhanced alkaline hydrogen evolution reaction. *Mater. Today Energy* **18**, 100513 (2020).

33. Shuai, C. et al. Nickel/cobalt bimetallic phosphides derived metal-organic frameworks as bifunctional electrocatalyst for oxygen and hydrogen evolution reaction. *J. Alloys Compd.* **847**, 156514 (2020).

34. Cheng, H. et al. $CoMo_2S_4$ with superior conductivity for electrocatalytic hydrogen evolution: elucidating the key role of Co. *Adv. Funct. Mater.* **31**, 2103732 (2021).

35. Jian, C., Cai, Q. & Liu, W. Three dimensional macroporous framework molybdenum disulfide-carbide heterojunction for highly efficient electrocatalytic hydrogen evolution at high current density. *Chem. Commun.* **57**, 11819–11822 (2021)

36. Huang, C. et al. N-doped Ni-Mo based sulfides for high-efficiency and stable hydrogen evolution reaction. *Appl. Catal. B: Environ.* **276**, 119137 (2020).

37. Huang, Z. et al. Selective selenization of mixed-linker Ni-MOFs: $NiSe_2$@NC core-shell nano-octahedrons with tunable interfacial electronic structure for hydrogen evolution reaction. *Appl. Catal. B: Environ.* **272**, 118976 (2020).

38. Xiong, J. et al. In situ engineering of double-phase interface in Mo/Mo_2C

heteronanosheets for boosted hydrogen evolution reaction. *ACS Energy Lett.* **3**, 341–348 (2018).

39. Huo, L. et al. Spatial confinement and electron transfer moderating Mo–N bond strength for superior ammonia decomposition catalysis. *Appl. Catal. B: Environ.*

40. Amama, P. B. et al. Role of water in super growth of single-walled carbon nanotube carpets. *Nano Lett.* **9**, 44–49 (2009).

41. Han, T. H., Huang, Y.-K., Tan, A. T. L., Dravid, V. P. & Huang, J. Steam etched porous graphene oxide network for chemical sensing. *J. Am. Chem. Soc.* **133**, 15264–15267 (2011).

42. Mathew, K., Sundararaman, R., Letchworth-Weaver, K., Arias, T. A. and Hennig, R. G. Implicit solvation model for density-functional study of nanocrystal surfaces and reaction pathways. *J. Chem. Phys.* **140**, 084106 (2014).

43. Parsons, R. The rate of electrolytic hydrogen evolution and the heat of adsorption of hydrogen. *Trans. Faraday Soc.* **54**, 1053–1063 (1958).

44. Nørskov, J. K. et al. Trends in the exchange current for hydrogen evolution. *J. Electrochem. Soc.* **152**, J23 (2005).

45. Greeley, J., Jaramillo, T. F., Bonde, J., Chorkendorff, I. B. & Norskov, J. K. Computational high-throughput screening of electrocatalytic materials for hydrogen evolution. *Nat. Mater.* **5**, 909–913 (2006).

46. Zheng, Y. et al. Hydrogen evolution by a metal-free electrocatalyst. *Nat. Commun.* **5**, 3783 (2014).
