# Supplementary material for: Water induced ultrathin Mo2C nanosheets with high-density grain boundaries for enhanced hydrogen evolution
Source: Nat Commun. 2022 Nov 24;13:7225. doi: 10.1038/s41467-022-34976-1 (PMC9700844; doi:10.1038/s41467-022-34976-1)
Supplement: Supplementary file 3 — Description of Additional Supplementary Files [file 41467_2022_34976_MOESM3_ESM.pdf]

Description of Additional Supplementary Files

File Name: Supplementary Data 1
Description: The atomic coordinates of the H adsorption on the fcc/hcp GB model to generate Fig. 6e.
